# Single‐Cell RNA Sequencing of Thyroid Tissues Reveals Pathogenesis of Graves' Disease

**DOI:** 10.1002/advs.202508449

**Published:** 2025-10-23

**Authors:** Xiaoyi Zhou, Jia Cong, Rongguang Peng, Dichen Yang, Chenchen Dong, Jing Xie, Jiqi Yan, Jie Kuang, Fubin Li, Leng Siew Yeap, Xiaoyan Xie, Haolong Yin, Rulai Han, Liyun Shen, Yulin Zhou, Guang Ning, Shu Wang, Weiqing Wang, Lei Ye

**Affiliations:** ^1^ Department of Endocrine and Metabolic Diseases Shanghai Institute of Endocrine and Metabolic Diseases Ruijin Hospital Shanghai Jiao Tong University School of Medicine (SJTUSM) Shanghai 200023 China; ^2^ Shanghai National Clinical Research Center for Metabolic Diseases Key Laboratory for Endocrine and Metabolic Diseases of the National Health Commission of the PR China Shanghai Key Laboratory for Endocrine Tumor Ruijin Hospital SJTUSM Shanghai 200023 China; ^3^ Department of Pathology Ruijin Hospital SJTUSM Shanghai 200025 China; ^4^ Department of General Surgery Ruijin Hospital SJTUSM Shanghai 200025 China; ^5^ Center for Immune‐Related Diseases at Shanghai Institute of Immunology Ruijin Hospital SJTUSM Shanghai 200025 China

**Keywords:** graves’ disease, immune cell atlas, single‐cell RNA sequencing, stress surveillance, Tph cells, γδ T Cells

## Abstract

Graves' disease (GD) is an autoimmune disorder primarily targeting the thyroid tissue. While major histocompatibility complex (MHC)‐dependent B cell activation and thyroid‐stimulating hormone receptor (TSHR) autoantibody production are central to GD, the intrathyroidal immune landscape remains largely unexplored. Through single‐cell RNA sequencing (scRNA‐seq), this work constructed a comprehensive immune cell atlas, revealing dominant IFN‐γ‐secreting CD4^+^ T cells, expanded T peripheral helper (Tph) cells, CD11c^+^ atypical B cells, and CD8^+^ effector T cells. Notably, stress‐surveilling γδ T/NK cells are enriched in GD. Thyroid follicular cells (TFCs) in GD exhibited a stressed phenotype, and in vitro functional assays showed that they promote γδ T cell activation and proliferation. γδ T cells may recruit conventional type 1 dendritic cells (cDC1) via XCL1/XCL2, suggesting a potential link to adaptive immune reorganization. These findings suggest an additional MHC‐independent pathway linking TFC stress to autoimmune activation via γδ T cells in GD pathogenesis.

## Graphical abstract

Using single‐cell RNA sequencing and TCR profiling, this study constructed an immune cell atlas in thyroid tissue of Graves’ disease (GD), revealing dominant IFN‐γ‐secreting CD4^+^ T cells, expanded Tph and CD11c^+^ B cells, and enriched T/NK cells. Notably, GD thyroid follicular cells (TFCs) activated γδ T cells, which recruited cDC1 cells, suggesting a mechanism linking TFC stress to autoimmune activation in GD pathogenesis.

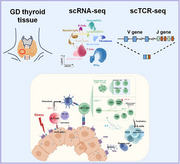

## 1 Introduction

GD is an autoimmune hyperthyroidism due to TSHR‐activating autoantibodies (TRAb), affecting ≈1.2% population globally.^[^ ^1^ ^]^ Both genetic and environmental factors (e.g., psychological stress, infections, iodine excess) contributed to GD pathogenesis.^[^ ^2^ ^]^ Unlike Hashimoto's thyroiditis (HT), a hypothyroid autoimmune thyroid disease (AITD), GD manifests as hyperplasia of thyroid follicular cells, hyperactive hormone synthesis, and increased vascularity. Approximately 40% of GD patients exhibit persistent or recurrent symptoms despite antithyroid drugs therapy, which indicates the diverse course of pathophysiology.^[^ ^3^ ^]^

In the classical GD model, TRAb is mainly produced by intrathyroidal B cells, while CD4^+^ helper T (Th) cells play a pivotal role through MHC‐restricted recognition of TSHR peptides on antigen‐presenting cells.^[^ ^4^ ^]^ But much remains unknown. Decades of research on GD‐related T cell compartments have yielded many findings, but remain controversial, particularly within thyroid tissue.^[^ ^1^, ^4^, ^5^ ^]^ Some studies suggest IL‐4‐secreting Th2 cells dominate in GD,^[^ ^6^, ^7^, ^8^, ^9^ ^]^ while others emphasize Th1 are predominant,^[^ ^10^, ^11^, ^12^ ^]^ particularly supporting by prevalence of IgG1‐type TRAb (Th1/IFN‐γ‐dependent) in human GD.^[^ ^13^ ^]^ Reduced peripheral regulatory T (Treg) cells, observed in most studies, are thought to underlie immune tolerance breakdown in GD, yet intrathyroidal Treg frequency remains conflicting.^[^ ^14^, ^15^, ^16^ ^]^ Beyond T/B cells, the roles of other immune populations in GD were poorly defined. These unresolved controversies and gaps in understanding underscore the necessity of comprehensive profiling of thyroid‐infiltrating cells of GD at the single‐cell level. Recent AITD spatial transcriptomics^[^ ^17^ ^]^ and TFC‐focused (excluding immune cells) scRNA‐seq study provided insights into TFC‐mediated antigen presentation and functions of stromal cells.^[^ ^18^ ^]^ However, a systematic single‐cell immune atlas of GD thyroid tissue remains absent, hindering a comprehensive understanding of its autoimmune pathogenesis and leaving the immune components largely unexplored.

Distinct from αβ T cells, γδ T cells utilize γ/δ‐chain TCRs to mount rapid, non‐MHC‐restricted responses to pathogens or tissue stress.^[^ ^19^, ^20^ ^]^ δ‐Chain variants determine huγδ T cell subsets. Vδ2 cells, abundant in blood, are more innate‐like, while Vδ2^−^ subsets include adaptive stress‐surveilling populations.^[^ ^21^ ^]^ In particular, tissue‐resident Vδ1 T cells undergo clonal expansion to detect cellular dysregulation markers.^[^ ^22^ ^]^ Endogenous stress molecules such as EPCR, ANXA2, and CD1 family members directly activate Vδ2^−^ T cells, driving localized immune reorganization and subsequent adaptive immune cascades.^[^ ^23^, ^24^, ^25^ ^]^ However, the role of γδ T cells in thyroid tissue remains unexplored.

In this study, we established a multi‐dimensional immune architecture of GD thyroid tissue through scRNA‐seq and TCR profiling. We confirmed that CD4^+^ T cells in GD patients are predominantly IFN‐γ‐secreting subsets, and identified Tph cells and CD11c^+^ atypical B cell subsets engaged in extrafollicular B cell responses. Importantly, our data reveal the increase of γδ T cells within the thyroid tissue of GD patients. We also provided evidence to hint that stress in thyroid follicular cells possibly activates them through up‐regulating ligands of γδ T cells, thereby potentially recruiting cDC1s to connect thyroid stress to adaptive immunity.

## 2 Results

### 2.1 Single‐Cell Profiling of Cell Composition in GD Thyroid Tissue

**Figure 1:**
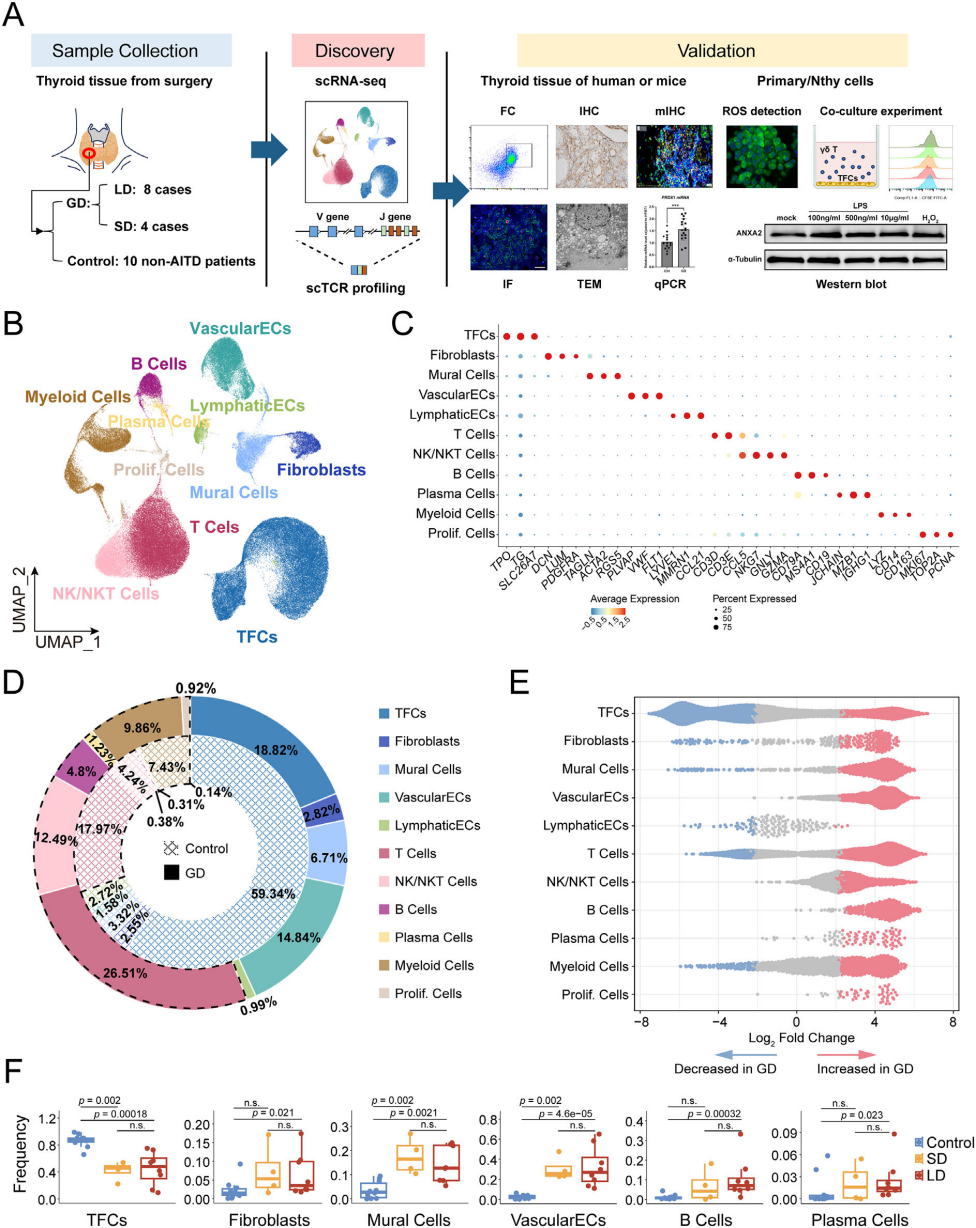
Single‐cell profiling of thyroid tissue samples from 12 GD and 10 control patients. A) Schematic overview of the experimental workflow. B) UMAP plots of 136,596 cells, derived from 22 samples, and cluster into eleven major cell groups. C) Dot plot showing the expression of classic marker genes for eleven cell clusters. D) Pie charts illustrate the proportions of 11 cell groups in GD and control, colored by UMAP identities. Checked inner ring (control) and outer ring (GD) highlight immune clusters with dashed outlines. E) Beeswarm plots illustrate the enrichment (red) or decrease (blue) of neighborhoods in GD for 11 cell types calculated using MiloR (FDR < 0.05). F) Proportions of 6 cell groups with significant proportional changes (immune and non‐immune cells calculated separately). Horizontal lines represent median values, and each dot signifies one sample. The *p*‐values are calculated with the Wilcoxon rank‐sum test.

To comprehensively resolve the cytological alterations in thyroid tissue of GD disease, we conducted scRNA‐seq on thyroid tissues from 12 GD patients and 10 non‐AITD controls. The GD cohort comprised 8 long‐duration (LD, >10 years post‐diagnosis) and 4 short‐duration (SD, <3 years) cases (**Figure** **1**A; Table S1, Supporting Information). After rigorous quality control, 136,596 high‐quality cells were retained for analysis (Table S1, Supporting Information). The αβ T cell receptor (TCR) sequences of individual cells were also profiled using single‐cell V(D)J sequencing. Unsupervised clustering analysis using uniform manifold approximation and projection (UMAP) identified 11 major clusters (Figure 1B; Figure S1A,B, Supporting Information). Residual batch effects are negligible after correcting with Harmony (Figure S1C,D, Supporting Information). Marker‐based annotation classified these clusters into five immune compartments (T cells, B cells, plasma cells, myeloid cells, and natural killer cells), thyroid follicular cells and four stromal compartments (vascular endothelial cells, lymphatic endothelial cells, fibroblasts, and mural cells comprising pericytes and smooth muscle cells), and one proliferative cluster (Figure 1B,C; Tables S2 and S3, Supporting Information).

In the control group, TFCs are predominant, accounting for 59.3%, while immune cells constitute 30.5%. In GD, the proportion of immune cells is increased to 55.8% (Figure 1D), among which B cells increased 720% (*p* = 2.6 × 10^−3^) (Figure 1E,F), displaying characteristic autoimmune signatures. Moreover, GD displayed significantly expanded stromal cells, particularly vascular endothelial cells, mural cells, and fibroblasts, reflecting the rich vascularity in GD (Figure 1E,F). Disease duration (LD vs SD) showed no significant compositional differences (Figure 1F; Figure S1E,F, Supporting Information).

### 2.2 Characterization of Conventional T and B Cell Populations in GD

#### 2.2.1 CD4^+^ T Cells Exhibited IFN‐γ Dominance and Extrafollicular B Helper Profile

**Figure 2:**
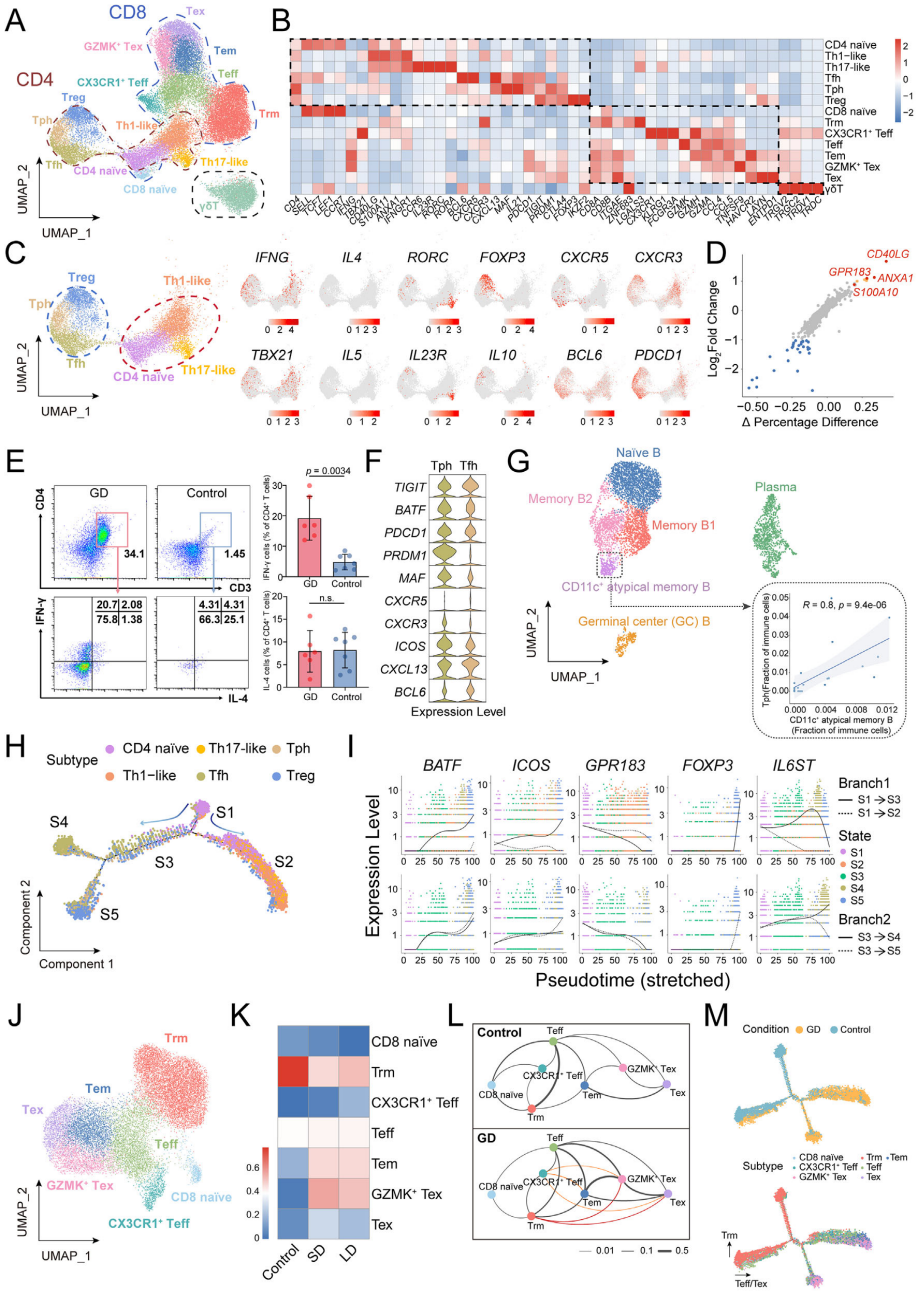
Enhanced IFN‐γ secretion and favoring B cell activation of CD4^+^ T, and expansion of CD8^+^ Effector T cells in GD thyroid tissue. A) UMAP of 32118 T cells clustered into fourteen subsets. B) Heatmap for expression of the representative marker genes used for T cell subsets identification. C) Six CD4^+^ T subsets segregate into pro‐inflammatory (red circle) and B helper (blue circle) groups. Marker gene expression is shown. D) Scatterplots showing DEGs between Th1‐like memory subset and other T cells. Genes with a log2FC ≥1 (7 upregulated and 29 downregulated) are highlighted. E) Flow cytometry quantification of the IFN‐γ and IL‐4 expression in intrathyroidal CD4^+^ T cell subsets of GD (N = 6) and control groups (N = 7). Quantification data (right) were expressed as mean ±s.d., two‐tailed Student's *t*‐test. F) Violin plots showing marker gene expression across Tph and Tfh subsets. G) UMAP distributions of the five B subsets and plasma cells (N = 5675). The inset box shows the positive correlation between the frequency of Tph and CD11c^+^ atypical B cells. Spearman's correlation test. H) The branched trajectory of CD4^+^ T cells. The arrow indicates pseudotime progression direction. I) Dynamic expression of representative genes at two branch points along pseudotime development. J) UMAP distributions of seven CD8^+^ T subsets (N = 18425). K) Heatmap showing clonal expansion in CD8^+^ T subsets across conditions. L) Clonal transitions between CD8^+^ T subsets. Edge thickness reflects shared clonotype ratios, and color lines highlight clonal transitions exclusive to GD. M) Pseudotime analysis of CD8^+^ T subsets colored by group (upper) or cell subtypes (lower).

T cells, comprising 50.1% of immune cells, were identified into 14 subsets based on classic markers: six CD4⁺, seven CD8⁺, and a striking subset of unconventional γδ T cells (**Figure** **2**A,B; Tables S2 and S3, Supporting Information).

CD4^+^ T subsets segregated into two groups (Figure 2C). One inflammatory effect group comprised naïve T cells, Th17‐like (*RORA^+^ RORC^+^* but lacking *IL17A/IL17F*), and Th1‐like memory cells defined by moderate Th1 markers (*IFNG*, *TBX21*) and high memory CD4^+^ T cell markers (*ANXA1, S100A1, CD40LG*) (Figure 2C; Figure S2C,D, Supporting Information).^[^ ^26^, ^27^ ^]^ These Th1‐like memory cells highly expressed *GPR183* (*EBI2*), a chemoattractant receptor that guides the positioning of CD4^+^ T cells and promotes their further differentiation into Tfh cells,^[^ ^28^, ^29^ ^]^ suggesting a potential role in supporting B cell response. Notably, a cluster of IL4^+^ CD4^+^ T cells was undetectable with universally low *IL4* levels across all cell groups in our data (Figure 2C; Figure S2E, Supporting Information), indicating attenuated Th2 activity in the GD microenvironment. The flow cytometry results confirmed a significant increase in CD4^+^IFN‐γ^+^ cells in the GD thyroid tissue, while CD4^+^IL‐4^+^ cells showed no significant change compared with the control group (Figure 2E).

Group 2 included three B helper T subsets: Tfh (*BCL6^+^CXCR5^+^PDCD1^hi^*), Tph (*CXCR5^−^CXCR3^+^ PDCD1^hi^*), and Tregs (*FOXP3* ^+^ *CTLA4* ^+^), all enriched in GD (Figure 2C; Figure S2A,B, Supporting Information). These Treg only expressed low levels of *IL10* and *PDCD1*, while they did express *IFNG, TBX21*, and high levels of *CXCR3* (Figure S2F, Supporting Information), suggesting a Th1‐like Treg phenotype with potentially impaired immunosuppressive function.^[^ ^30^, ^31^ ^]^ Moreover, Tph and Tfh cells also broadly expressed *IFNG* (Figure 2C; Figure S2F, Supporting Information).

Notably, we identified *PDCD1* ^hi^ *CXCR5* ^−^ Tph^[^ ^32^, ^33^ ^]^ cells enriched in thyroid tissue of GD for the first time (Figure 2F; Figure S2A,B, Supporting Information), this enrichment directly links to extrafollicular B cell activation, which may explain the minimal germinal centers observed in GD. These cells highly expression of *MAF* and *PRDM1*, and shared *CXCL13* and *ICOS* expression with Tfh (Figure 2F).

Correspondingly, five B cell subsets were identified, including CD11c^+^ atypical B cells characterized by *CD11c*, *TBX21*, and *FCRL5* expression^[^ ^34^ ^]^ (Figure 2G; Figure S3A, Supporting Information). These cells mediating extrafollicular B cell activity were nearly exclusive in GD (Figure S3B–D), enabling rapid differentiation into antibody‐secreting cells.^[^ ^35^ ^]^ Their abundance strongly correlated with Tph cells (*r* = 0.8, Figure 2G), suggesting Tph‐mediated promotion of this pathogenic B cell subset.

Taken together, GD thyroid CD4^+^ T cells exhibited two properties: (1) a Th1‐like microenvironment with low *IL4* and absent Th2 clusters, dominated by *IFNG*‐expression subsets; and (2) a B‐cell‐facilitating milieu driven by expanded Tph/Tfh and GPR183^+^ Th1‐like cells, supporting pathogenic extrafollicular B cell activation.

Next, we investigated how the CD4^+^ T cell pattern formed. Pseudotime trajectory analysis revealed naïve CD4^+^ T cells (State 1) bifurcated into Th1/Th17 like‐dominant State 2 and a transitional State 3. Subsequent bifurcation separated Treg‐predominant State5 and Tph/Tfh/Treg‐mixed State 4 (Figure 2H; Figure S2H–K, Table S4, Supporting Information). Key regulators at the first bifurcation included *GPR183, BATF*, and *ICOS* (Figure 2I). Their dynamic expression along State 2/3 trajectories suggested *GPR183* associates with Th1/Th17‐like differentiation, while *BATF* and *ICOS* may facilitate Tph/Tfh/Treg lineage transitions. *FOXP3* expression became prominent during State 5. *IL6ST* (a co‐receptor for IL‐6) exhibited strikingly dynamic expression at both bifurcations, implicating its regulatory role in lineage commitment (Figure 2I). MemB1 and partial naïve B serve as major IL6 sources (Figure S3E, Supporting Information), indicating reciprocal B cell modulation of T cell polarization patterns. These data suggested IL6 as a key contributor to the CD4^+^ T cell pattern formation in GD. Notably, IL‐6 receptor monoclonal antibody tocilizumab has emerged as a second‐line therapy for Graves' orbitopathy.^[^ ^36^ ^]^

#### 2.2.2 Expansion ofCD8^+^ T Cell Subsets with Effector Properties in GD Thyroid Tissue

In the CD8^+^ T cell compartment, multiple subsets such as naïve cells and effector populations—including CX3CR1^+^ Teff,^[^ ^37^ ^]^ Teff, Tem, and GZMK^+^ Tex—were significantly more abundant in GD patients than in controls (Figure 2J; Figure S4A, Supporting Information). Moreover, the proportion of *ITGAE^+^ ZNF683^+^ CXCR6^+^* Trm cells^[^ ^38^ ^]^ was notably higher in control and negatively correlated with TRAb levels (Figure S4B, Supporting Information), potentially implicating Trm cells in autoantigen clearance.

Expansion of effector CD8^+^ T cell subsets was further corroborated by scTCR profiling. Effector subsets (Teff, Tem, GZMK^+^ Tex) showed clonal expansion in GD thyroid tissues across SD and LD (Figure 2K). Shared TCR clonotypes indicated Trm and CX3CR1^+^ Teff cells drove effector expansion in GD via differentiation into effector populations (Figure 2L). Pseudotime trajectory analysis also indicated enhanced CD8^+^ effector differentiation in GD, showing control CD8^+^ T cells predominantly became Trm, while GD CD8^+^ T cells progressed toward Teff, Tem, and Tex subsets (Figure 2M; Figure S4C, Supporting Information). The evaluation of cytotoxic T cells suggests chronic autoimmune activation and disrupted immune homeostasis in GD thyroid tissues.

### 2.3 Expansion of Unconventional T Cells: γδ T Cells in GD are Linked to Stress‐Surveillance and Immune Reorganization

**Figure 3:**
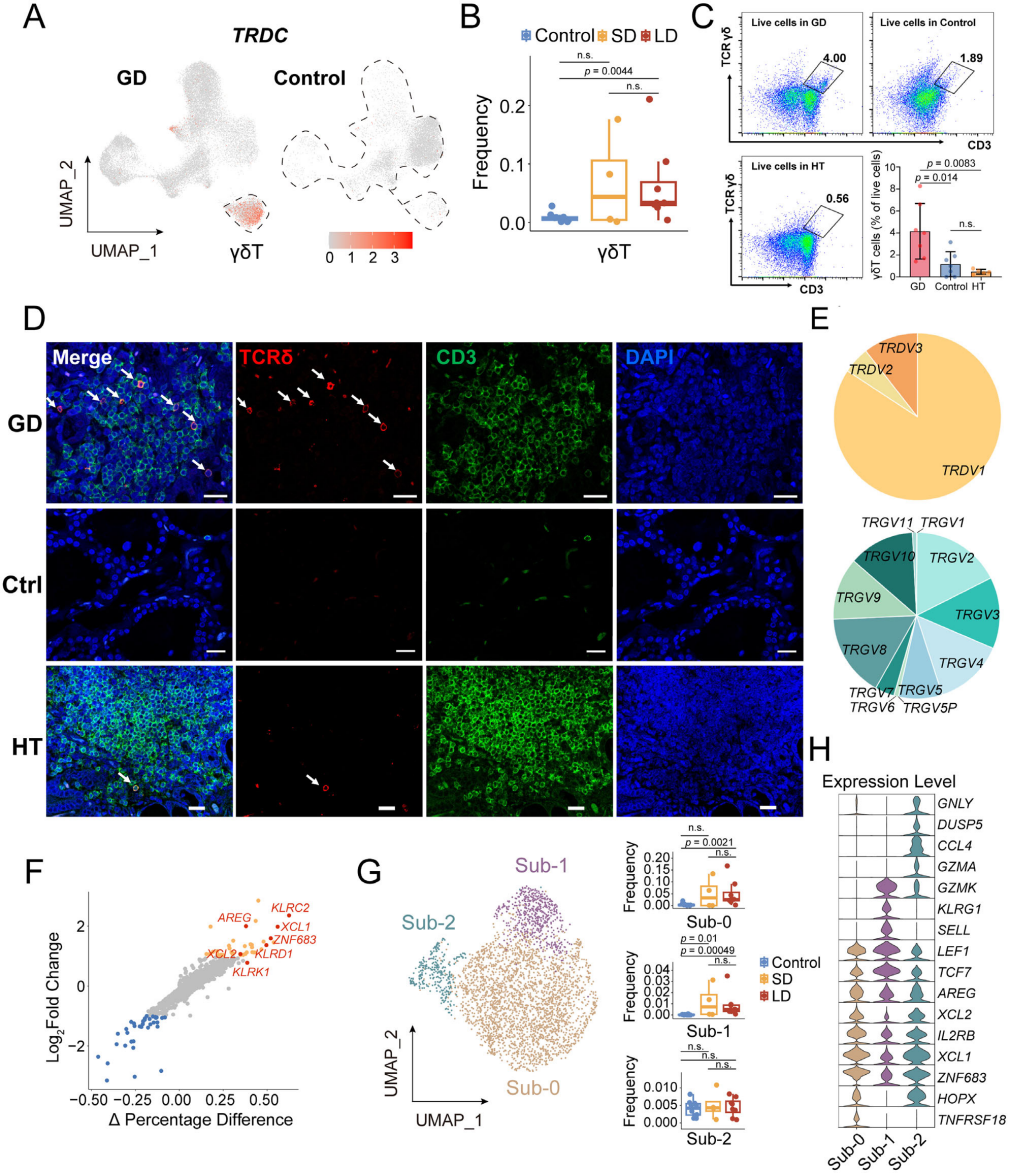
γδ T Cells expand in GD Thyroid Tissue and exhibit Tissue Repair and Immune Reorganization Signatures. A) TRDC (TCR δ Chain Constant) marks GD‐enriched γδ T Cell Subsets. B) Elevated proportions of γδ T cell subsets among immune cells in GD compared to control. Wilcoxon rank‐sum test. C) Flow cytometry quantification of γδ T cell subsets in thyroid tissues from GD (N = 7), HT (N = 5), and control (N = 7) patients. Quantification data were expressed as mean ± s.d. Two‐tailed student's *t*‐test. D) Immunofluorescence staining for TCRδ (red) and CD4(green) showing the distribution of γδ T cells in GD, HT, and control (Ctrl). The arrows indicate γδ T cells co‐stained with TCRδ and CD4, bar = 20 µm. E) Composition ratios of subtypes of γ and δ chains in γδ T cells, with predominant Vδ1 subtype and diverse γ chain combinations. F) Scatterplots showing DEGs between γδT cells and other T cells. Genes with a log2FC ≥1 (37 upregulated and 35 downregulated) are highlighted. G) UMAP of three γδ T subpopulations (N = 3484) under high resolution (left), and their proportions across different groups (right). Wilcoxon rank‐sum test. H) Violin plots showing representative genes across γδ T subpopulations.

Intriguingly, γδ T cells were strikingly expanded in GD thyroid tissue relative to control tissues (**Figure** **3**A,B). γδ T cells, often described as “innate‐like” T cells, serve as an interface bridging innate and adaptive immunity.^[^ ^39^ ^]^ To assess disease specificity, we also analyzed HT, a distinct AITD, using a published scRNA‐seq dataset,^[^ ^40^ ^]^ and detected no γδ T cell cluster in HT lesions (Figure S5A, Supporting Information). Flow cytometric quantification also demonstrated the elevation of thyroid tissue γδ T cells in GD patients compared to both control and HT counterparts (Figure 3C). Immunofluorescence analysis revealed that these cells were scattered and distributed in the area of local lymphocytic infiltration in GD thyroid tissue, whereas scarce in normal tissues and only minimally detected in HT specimens (Figure 3D). We further investigated thyroid tissues from GD patients with very short disease duration (<2 months post‐diagnosis). Immunohistochemical analysis revealed that the density of γδ T cells was already significantly elevated in these early GD samples and comparable to that in the LD group (Figure S5B, Supporting Information), suggesting that γδ T cell expansion occurs early in GD and is not solely a consequence of chronic disease or long‐term treatment. These results establish γδ T cell accumulation as a GD‐specific immunological feature in the thyroid, implicating their potential role in pathogenic mechanisms.

Subsequently, we assessed the features of thyroid γδ T cells. Analysis of TCR γδ chain‐encoding genes demonstrated a predominant Vδ1 subtype in thyroid tissue, accompanied by diverse γ chain combinations (Figure 3E). Vδ1^+^ γδ T cells exhibit adaptive features that distinguish them from the more innate‐like Vδ2 subset and are implicated in tissue‐specific immunosurveillance.^[^ ^19^, ^20^ ^]^ Notably elevated molecules included tissue‐residency marker *ZNF683*, activation markers *CD69*, and “adaptive” NK‐related receptors *KLRC2*(*NKG2C*),^[^ ^41^ ^]^ *KLRD1*, and *KLRK1*(*NKG2D*)^[^ ^42^ ^]^ (Figure 3F; Figure S5C,D, Supporting Information). Previous studies demonstrated that tissue‐resident Vγ5Vδ1 T cells activated through NKG2D ligands play pivotal roles in stress molecule detection and local immune reorganization.^[^ ^43^ ^]^ The thyroid γδ T cells also showed high expression of *XCL1* and *XCL2*, chemokines critical for recruiting conventional type 1 dendritic cells (cDC1),^[^ ^44^ ^]^ and *AREG*, a growth factor associated with tissue repair.^[^ ^45^ ^]^ Although γδ T cells are generally reported to produce Th1/Th2/Th17 cytokines such as IFN‐γ, TNF, IL‐4, and IL‐17,^[^ ^46^, ^47^ ^]^ γδ T in thyroid tissue exhibited a unique *IFNG^mid^ TNF^low^ IL‐4^−^* *IL‐17^−^* phenotype (Figure S5C, Supporting Information), suggesting the attenuated inflammatory activity.

To resolve functional heterogeneity among thyroid γδ T cells, we performed re‐clustering at 0.2 resolution and identified three distinct subpopulations (Figure 3G; Table S5, Supporting Information). Sub‐1 displayed elevated naïve markers *SELL* and *LEF1*, indicative of a less differentiated state. Nearly all γδ T cells from control samples were confined to Sub‐2, a minor cluster highly expressed granzymes and *GNLY*, suggesting Sub‐2 may represent a cytotoxic subset involved in baseline immunosurveillance (Figure 3G,H). The dominant population Sub‐0 (accounting for 90% of thyroid γδ T cells) displayed minimal expression of effector or cytotoxic molecules but was enriched for the tissue‐repair factor *AREG* and the chemokines *XCL1* and *XCL2*, alongside moderate IFN‐γ levels (Figure 3H; Figure S5C, Supporting Information). Functionally, the dominance of Sub‐0 suggests that in GD, *AREG* expression points to a directly contribution in tissue repair, while XCL1/XCL2 likely facilitates cDC1 recruitment, thereby linking innate stress detection to immune response organization.

Collectively, these results suggest the function of thyroid γδ T cells in stress‐surveillance: coordinating protective tissue repair and immune cell recruitment to reorganize the local immune compartment.

### 2.4 Cooperative γδ T and NK Cells Drive Recruitment of cDC1 in GD Pathogenesis

**Figure 4:**
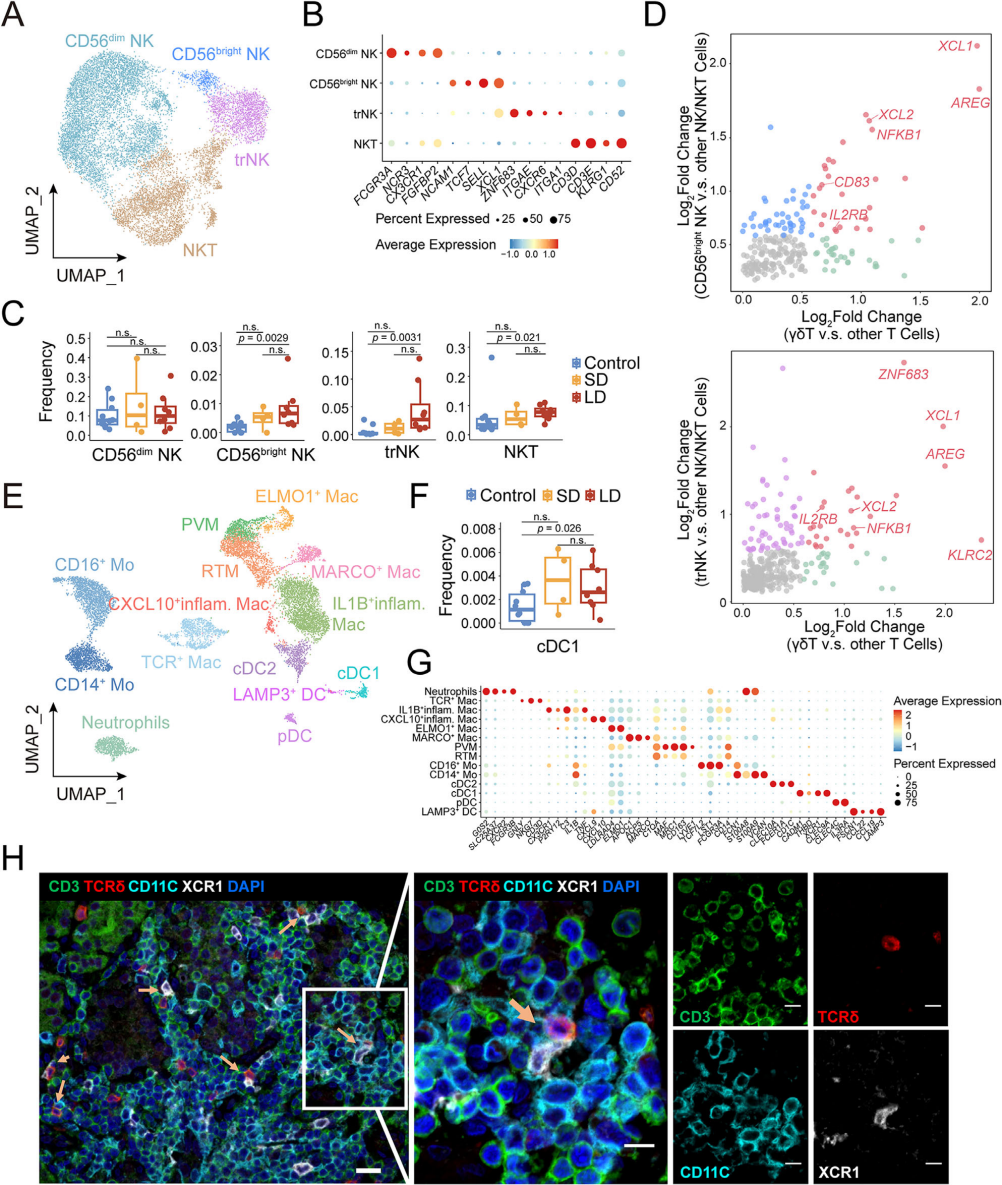
γδ T/NK Synergy in cDC1 Recruitment via XCL1. A) UMAP of three NK and one NKT clusters (N = 13104). B) Dot plot showing the expression of classic marker genes for NK/NKT clusters. C) Proportions of NK/NKT subsets among immune cells in different groups. Wilcoxon rank‐sum test. D) Scatterplots showing shared DEGs between CD56^bright^ NK cells (upper) and trNK cells (lower) with γδ T cells. Each dot represents one gene, with shared genes colored red, γδ T‐specific genes colored green, and NK cell‐specific genes colored either blue or purple. E) UMAP distribution of fourteen myeloid cell subsets (N = 12300). F) The elevated proportions of cDC1 among immune cells in GD compared to control. One‐tailed Student's *t*‐test. G) Dot plot showing the expression of classic marker genes for myeloid cell sub‐clusters. H) Representative polychromatic immunostaining showing the distribution of CD3^+^TCRδ^+^ γδ T cells and CD11c^+^XCR1^+^ cDC1. Arrows indicate adjacent γδ T cells and cDC1 cells. The right panel (bar = 10 µm) is a magnified view of the white box in the left panel (bar = 20 µm).

Analysis of innate immune compartments revealed coordinated activation in GD. We identified two classical circulating NK (cirNK) subsets: CD56^dim^ and CD56^bright^ NK, one distinct *ZNF683^+^ ITGAE^+^ CXCR6^+^* trNK^[^ ^48^, ^49^ ^]^ cluster, and a CD3^+^ NKT subset (**Figure** **4**A,B; Figure S6A,B, Table S6, Supporting Information). CD56^bright^ NK, trNK, and NKT subsets were all significantly expanded in GD (Figure 4C). Notably, GD‐enriched trNK and CD56^bright^ NK exhibited significant co‐upregulation with γδ T cells of effector molecules *XCL1*, *XCL2*, and *AREG*, accompanied by shared overexpression of *KLRC2* and *IL2RB* (Figure 4D; Figure S6C, Supporting Information). NK cells are also key sensors in stress‐surveillance. This common molecular signature suggests their functional synergy with γδ T cells in GD pathogenesis.

Myeloid cells comprise 14 subsets: four dendritic cell clusters, seven macrophage (Mac) subsets, CD14^+^/CD16^+^ monocytes, and neutrophils (Figure 4E,G). Thyroid Mac displayed functional diversity, encompassing two M2 Mac: *CD163^+^C1QA^+^MRC1^+^* resident tissue Mac (RTM), and *LYVE^+^* vascular‐associated Mac (PVM), alongside phagocytosis‐associated clusters *MARCO^+^* Mac and *ELMO^+^* Mac, and pro‐inflammatory subsets *IL1B^+^* Mac and *CXCL10* ^+^Mac^[^ ^50^, ^51^ ^]^ (Figure 4G; Figure S6F, Supporting Information).

Of particular interest, cDC1 cells expressing XCR1 (receptor for XCL1/XCL2) were increased in GD (Figure 4F; Figure S6D, Supporting Information), paralleling the expansion of XCL1/XCL2‐producing γδ T/NK cells. Spatial analysis via multiplex immunofluorescence reveals proximity between TCRD^+^CD3^+^ γδ T cells and CD11c^+^XCR1^+^ cDC1, indicative of γδ T‐mediated cDC1 recruitment (Figure 4H). As critical antigen‐presenting cells for CD8⁺ T cell activation,^[^ ^52^, ^53^ ^]^ cDC1 expansion could enhance cytotoxic T cell responses in GD. Consistent with this, there is a positive correlation between the local proportions of γδ T and GZMK IHC positive cells within lymphocytic infiltrates (Figure S6G, Supporting Information). Taken together, these findings imply a stress‐surveillance axis linking innate, myeloid, and adaptive immunity, centered on γδ T/NK‐derived signals and cDC1‐mediated immune reorganization in GD.

### 2.5 TFCs in GD Exhibit a Heightened Oxidative Stress State

**Figure 5:**
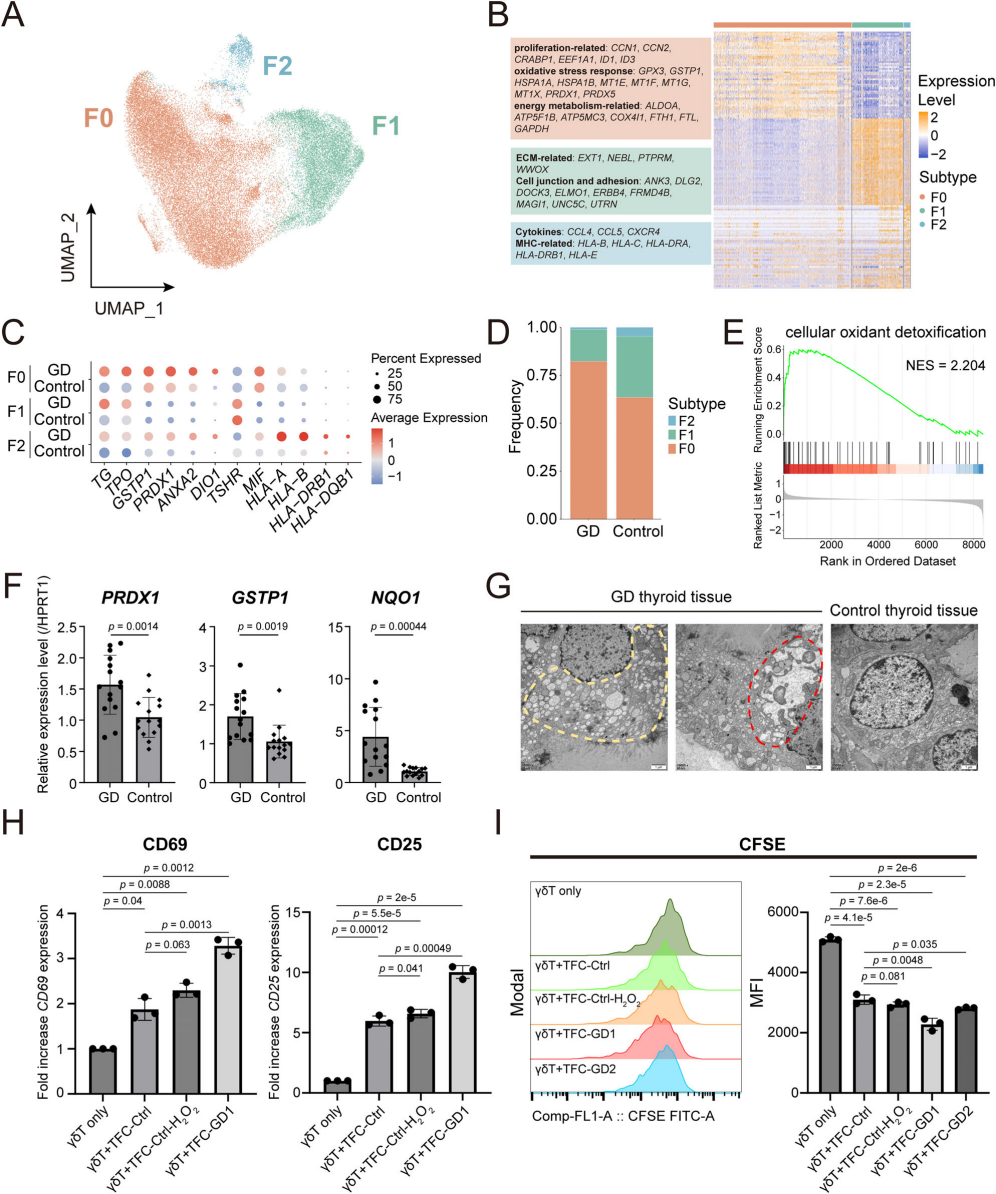
TFCs in GD sustain an elevated stress state. A) UMAP of three TFC subsets (N = 45161). B) Heatmap illustrating the expression levels of the top 50 genes in each TFC subset. Representative genes for each subset are listed in the left box, colored according to their UMAP cluster identities. C) Dot plots displaying the elevated expression of thyroid hormone synthesis, stress response, and antigen presentation‐related genes in three TFC subsets across GD and control. D) Three TFC subpopulation distributions across the GD and control groups. E) GSEA map showing the enrichment of cellular oxidant detoxification genes in TFCs of GD compared to control. F) Representative oxidative stress‐related genes show upregulated relative expression levels in GD (N = 15) compared to control (N = 15). Data are representative of three independent experiments and expressed as mean ± s.d., two‐tailed Student's *t*‐test. G) Transmission electron microscopy (TEM) examination illustrating the abnormal ER in GD, including vesiculation (yellow frame) and dilation (red frame). bar = 1µm. H) Proportion changes of activation markers CD69^+^ and CD25^+^ in γδT Cells after 48‐h co‐culture with primary TFCs from GD or control donors. Data (mean ± s.d. of three technical replicates) from one representative donor are shown, with similar results observed in two other independent donors. Two‐tailed Student's *t*‐test. I) CFSE‐labeled γδ T cells were co‐cultured with primary TFCs for 60 h and then analyzed by flow cytometry. Representative histograms (left) and MFI (right) show CFSE dilution. Data in the bar graph are expressed as mean ± s.d. of three technical replicates. Similar results were observed in three independent donors. Two‐tailed Student's *t*‐test.

Next, we examined thyroid follicular cells to explore potential triggers of pathogenic immunity in GD. TFCs are divided into three subsets (**Figure** **5**A). The F0 subset shows high expression of proliferation‐associated genes such as *ID3*, *EEF1A1*, and *CCNs*, metabolic markers including *ALDOA* and *GAPDH*, and stress‐response genes like *PRDX1*, *GSTP1*, *HSPA1A*, and *GPX3*, suggesting rapid proliferation, metabolic hyperactivity, and stress adaptation (Figure 5B). The F1 subset expressed adhesion and extracellular matrix genes, while the F2 subset displayed increased cytokines (*CCL4*, *CCL5*) and MHC class I/II molecules (Figure 5B). Although overall TFC proportion decreased due to the expansion of vascular endothelial cells (Figure S7A,B, Supporting Information), TFC subsets in GD exhibited upregulation of thyroid hormone synthesis genes (*TPO*, *DIO1*, *TG*), *MIF*, and MHC molecules (Figure 5C), corroborating previous findings.^[^ ^17^, ^18^ ^]^

The F0 subset, characterized by high expression of stress‐response genes, represents the predominant population within GD thyroid tissue (Figure 5D), Consistent with this observation, gene set enrichment confirmed oxidant detoxification and stress response pathways were heightened in GD TFCs (Figure 5E; Figure S7C, Table S7, Supporting Information). qRT‐PCR further confirmed significant upregulation of genes linked to cellular defense against stress and reactive oxygen species (ROS), including *PRDX1*, *GSTP1* , and *NQO1*, in comparison to the control group (Figure 5F). Additionally, GD TFCs exhibit abnormal endoplasmic reticulum (ER) morphology, characterized by dilation and vesiculation, corroborating their heightened stress status (Figure 5G). These results confirmed that TFCs in GD sustain a state of elevated stress. To further investigate the functional impact of GD TFCs on γδ T cells, we co‐cultured primary TFCs from GD donors with Vδ1⁺ γδ T cells. Compared to TFCs from control donors, GD TFCs significantly enhanced the activation and proliferation of γδ T cells (Figure 5H,I). This stimulatory effect, though weaker, was also observed when control donor TFCs were pre‐treated with H_2_O_2_ to mimic oxidative stress. Together, these results confirmed that TFCs in GD sustain a state of elevated stress, and further suggest that this stressed phenotype may underlie their enhanced capacity to activate stress‐surveilling γδ T/NK cells.

### 2.6 GD TFCs Exhibited Elevated γδ T Cell Ligands

**Figure 6:**
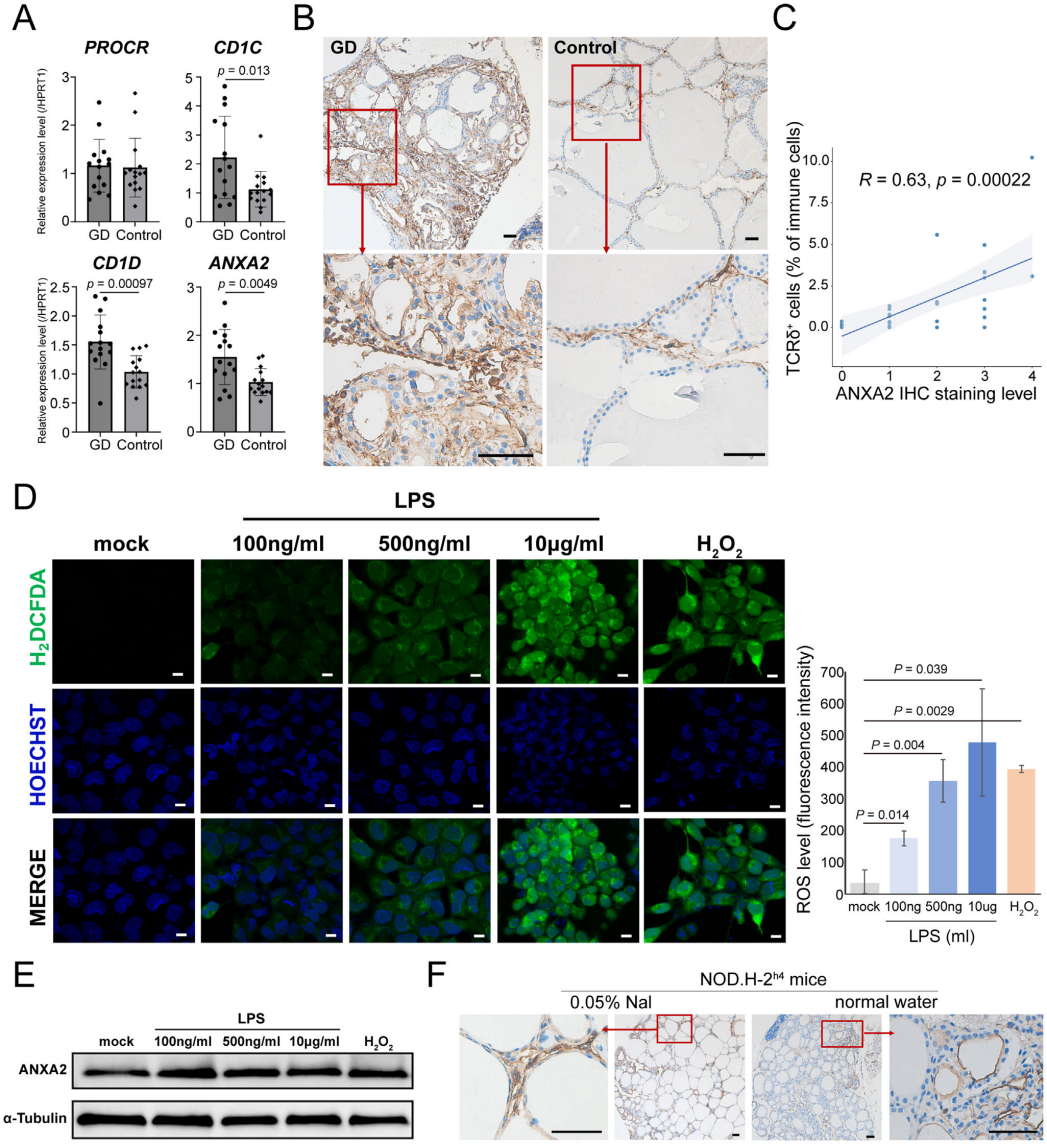
TFCs in GD exhibited elevated stress ligand ANXA2. A) The expression levels of known ligands of Vδ1 T cells detected by qRT‐PCR in GD (N = 15) and control (N = 15). Data were expressed as mean ± s.d., two‐tailed Student's *t*‐test. B) Representative ANXA2 IHC staining in GD and control thyroid tissues. bar = 50 µm. C) The correlation between ANXA2 staining level and the proportion of TCRδ‐positive cell in local lymphocytic infiltrates. Spearman's correlation test. D) The intracellular ROS levels in Nthy cell line under LPS or H_2_O_2_ treatment, illustrated by H_2_DCFDA (green) and Hoechst (blue) for nuclear staining. bar = 10 µm. Quantification data are expressed as mean ± s.d., two‐tailed Student's *t*‐test. E) Western blot showing elevated ANXA2 levels in Nthy under LPS or H_2_O_2_ treatment. F) IHC showing the expression of ANXA2 in NOD.H‐2^h4^ mice with NaI added or normal water. bar = 50 µm.

The high‐stress state of TFCs in GD evokes associations with stress‐surveillance γδ T cells and NK cells. Therefore, we detected the expression of currently known intracellular stress surveillance‐associated γδ T cell ligands^[^ ^21^, ^54^ ^]^ and observed a significant upregulation of *CD1D* and *ANXA2* in GD thyroid tissue compared to the control (**Figure** **6**A). *ANXA2* encodes a Ca^2^⁺‐regulated phospholipid‐binding annexin protein,^[^ ^55^ ^]^ which is upregulated and translocates to the membrane under oxidative stress and directly binds to γδ T cell receptors to activate these cells.^[^ ^25^ ^]^ Immunohistochemical analysis demonstrated regionally heterogeneous upregulation of ANXA2 TFCs in GD patients, with staining intensity positively correlating with the local proportion of γδ T cells within lymphoid infiltrates (Figure 6B,C; Figure S7D, Supporting Information).

To investigate whether cellular stress directly induces ANXA2 upregulation in TFCs, we treated thyroid follicular cell line Nthy with LPS or H_2_O_2_, as infection and stress are both risk factors for GD. These treatments increased intracellular ROS levels and ANXA2 protein level (Figure 6D,E). Furthermore, since high iodine is a risk factor for GD, we tested the effect of a high iodine diet in NOD.H‐2^h4^ autoimmune thyroiditis mice model.^[^ ^56^, ^57^ ^]^ We found that ANXA2 expression in TFCs was also upregulated compared to controls (Figure 6F). These findings suggest that stress‐inducing stimuli in TFCs promote upregulation of endogenous stress molecules like ANXA2, which may activate immune‐surveillance γδ T cells, thereby driving immune cell repertoire remodeling, eventually drive both the initial development or clinical exacerbation of GD.

### 2.7 Thyroid Stromal Cells in GD Displayed Limited Chemotaxis and Enhanced Angiogenesis

**Figure 7:**
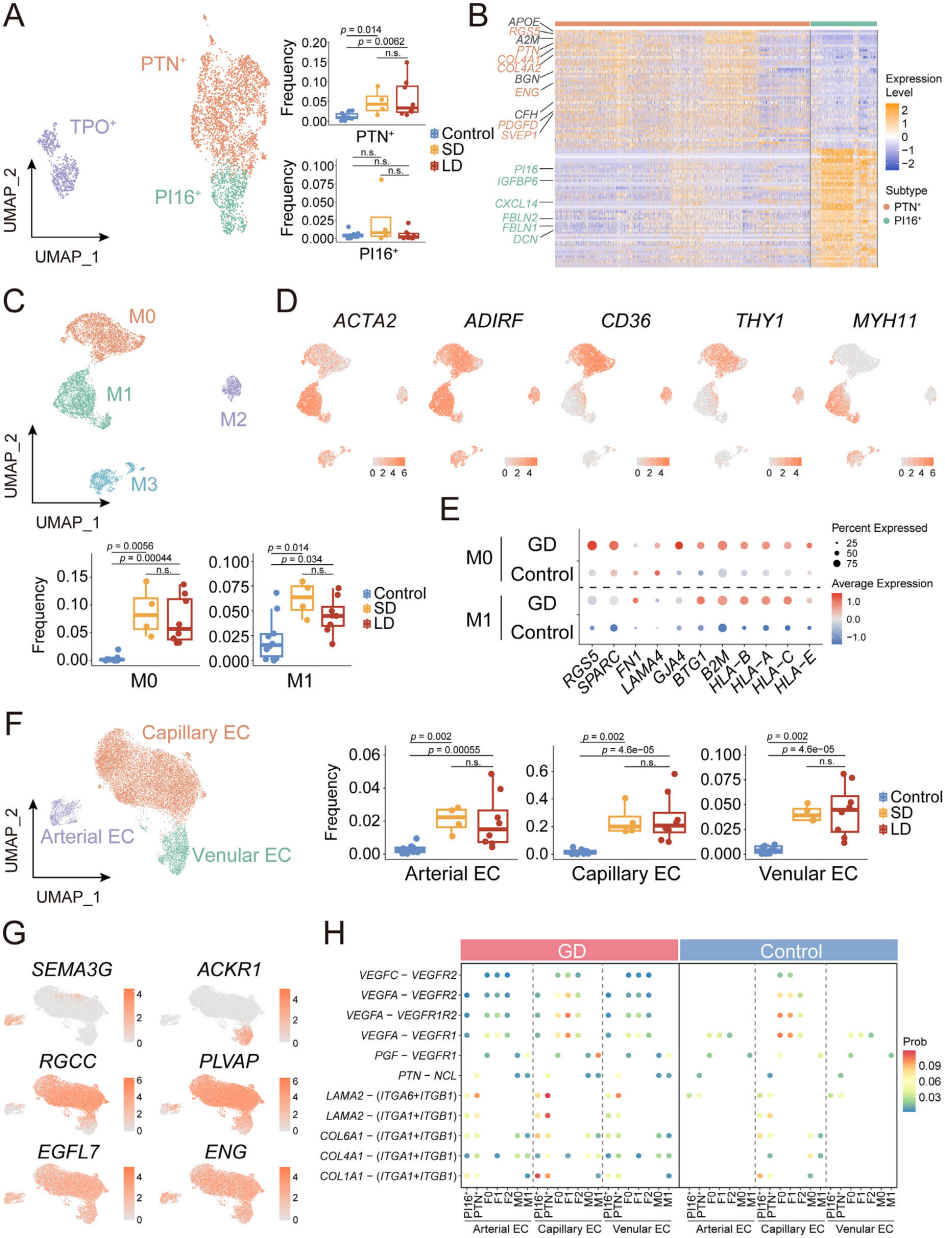
Enhanced Angiogenic Properties of Stromal Cells in GD. UMAP distribution and proportions (in non‐immune cells) of A) fibroblasts subsets (N = 3718), C) mural cells subsets (N = 7542), and F) vascular endothelial cell subsets (N = 13909). B) Heatmap of the expression levels of the top 50 genes in each fibroblast subset. D) Representative marker gene expression across mural cell subsets. E) Dot plots displaying the upregulation of pro‐angiogenic and MHC‐I genes in M0 and M1 in GD compared to control. G) Representative marker gene expression across vascular endothelial cell subsets. H) Cell communication from TFC/other stromal cells (sending) to vascular endothelial cells (receiving). Only the significant GD‐specific interactions, as compared to the control, are displayed.

We next characterized the stromal landscape, which underpins the architectural and functional changes in the GD thyroid tissue. Fibroblasts were categorized into three subsets (**Figure** **7**A,B): the PI16^+^ subset (*IGFBP6* ^+^ *PI16* ^+^ *CXCL14* ^+^),^[^ ^58^ ^]^ aligning with inflammatory associated fibroblasts in AITD^[^ ^17^ ^]^ and the F2 subset in HT;^[^ ^40^ ^]^ an extracellular matrix (ECM) remodeling associated PTN^+^ subset, expanded in GD (Figure S8A, Supporting Information); and a TPO^+^ subset, also identified in previous study.^[^ ^17^ ^]^ Interestingly, chemokines *CCL2*, *CCL19*, *CCL21*, and *CXCL9*, which are highly expressed in HT fibroblasts,^[^ ^40^ ^]^ are barely detectable in GD fibroblasts (Figure S8B, Supporting Information), potentially correlating with the relatively reduced lymphoid infiltration observed in GD compared to HT.

Mural cells, comprising pericytes and smooth muscle cells, were classified into four ACTA2^+^ subsets^[^ ^59^ ^]^ (Figure 7C). Among these, M0 subset expressed pericyte markers *CD36* and *THY1*, while M1 showed elevated smooth muscle cell marker *MYH11*, mirrors two *ACTA2+* subpopulations in HT^[^ ^40^ ^]^ and being significantly enriched in GD (Figure 7D; Figure S8C, Supporting Information). Interestingly, these GD‐enriched cell subsets also lacked the chemokine signature seen in HT^[^ ^40^ ^]^ (Figure S8D, Supporting Information), instead upregulating pro‐angiogenic genes such as *RGS5*, *SPARC*, *GJA4*, and MHC I molecules (Figure 7E). GD‐specific differentially expressed genes in M1 were significantly enriched in antigen presentation and ECM organization‐related processes (Figure S8E, Table S8, Supporting Information).

Vascular endothelial cells were classified into three subtypes (Figure 7F,G): *SEMA3G* ^+^ arterial ECs, *RGCC* ^+^ *FTL* ^+^ capillary ECs, and *ACKR1* ^+^ venous ECs,^[^ ^60^ ^]^ all of which were markedly expanded in the GD tissue (Figure S8F, Supporting Information). Capillary ECs predominated, consistent with previous spatial transcriptomics studies reporting their high expression of vascular permeability‐related genes *PLVAP* ^[^ ^17^ ^]^ and angiogenesis‐associated genes (*UQCR11*, *UQCRQ*, *EGFL7*, and *ENG*) (Figure S8G, Supporting Information). However, compared to HT, ECs in GD thyroid tissue exhibited significantly lower expression of high endothelial venule (HEV)‐associated genes (*SELP*, *IL33*, *SELE*, *MADCAM1*)^[^ ^40^, ^61^ ^]^ (Figure S8G, Supporting Information). Cell communication analysis revealed significantly enhanced interactions in GD tissues between *Pi16^+^* and *PTN^+^* fibroblasts and Mural M0 subsets with ECs compared to controls (Figure S8H, Supporting Information). These interactions were mediated by *LAMA2*, *COL6A1*, and *COL1A1*, which stabilize nascent vasculature through basement membrane assembly and ECM remodeling, alongside *VEGFA*‐driven endothelial proliferation/migration and *PTN*‐mediated PI3K/AKT signaling activation to potentiate angiogenesis (Figure 7H, Table S9, Supporting Information). These results align with the increased thyroid vascularity in GD, which manifests as the characteristic ‘thyroid inferno’ pattern on color Doppler ultrasonography.

Finally, we investigated cell types relevant to the genetic risk loci of GD. By integrating 44 genes from 28 GD‐associated loci reported in prior GWAS and linkage analyses^[^ ^2^, ^62^, ^63^, ^64^ ^]^ (Table S10, Supporting Information), we identified significant SNP‐heritability enrichment in 14 cell subtypes (Figure S9, Supporting Information). These included CD8+ T cell subsets with effector properties, *CXCL10+* inflam. MAC, cDC1, and high‐stress F0 thyroid follicular cell clusters, highlighting the genetic basis of these cellular mechanisms in GD.

## 3 Discussion

Thyroid is the primary affected organ in GD. To advance understanding of GD, we performed scRNA‐seq on thyroid tissue and generated a high‐resolution cellular atlas, with refined T cell subtyping. Previous studies^[^ ^5^, ^6^, ^7^, ^8^, ^9^, ^10^, ^11^, ^12^ ^]^ on CD4^+^ T cell Th1 or Th2 polarization have reported mixed findings, partly due to methodological differences, including reliance on peripheral blood or murine models, limited thyroid tissue analyses, and conventional Th1/Th2 definitions based on solely gene marker. scRNA‐seq data allow us to more accurately define cell types by utilizing multi‐gene transcriptional signatures, revealing that within the GD thyroid, there is a Th1‐like memory subset with moderate IFNG and TBX21 expression. Meanwhile, multiple CD4^+^ subsets (Treg, Tfh, Tph) also expressed IFNG. However, since our GD thyroid samples were obtained from surgically treated patients with >1 year of therapy, they may not reflect early‐stage GD pathogenesis. Further analysis of paired peripheral blood mononuclear cells (PBMCs) and comparison with PBMCs from untreated, newly diagnosed GD patients may clarify disease progression dynamics. Elucidating untreated GD thyroid tissue will require collaborative efforts to address sampling challenges. Additionally, this study is limited by the low number of male samples, a common challenge in thyroid disease research due to its higher prevalence in females.^[^ ^4^, ^65^ ^]^ Although our preliminary analysis showed no evidence of sex‐biased clustering, this aspect warrants further investigation in larger, sex‐matched cohorts.

Given the diverse course of GD,^[^ ^3^ ^]^ we enrolled 4 SD and 8 LD cases. Most immune subsets exhibited shared trends in SD and LD compared to controls, but LD showed more pronounced changes—particularly in CD4^+^ and CD8^+^ effector T cells, indicative of chronic autoimmune activation. However, γδ T and cDC1 subsets showed no stronger trends in LD than SD, implying common pathogenic mechanisms across disease stages. Limited sample size precludes deeper exploration of SD/LD differences; future studies with larger cohorts are needed to clarify GD's temporal dynamics.

We found significant γδ T subsets in GD thyroid tissue. These cells, primarily of the Vδ1‐type, which were reported as more adaptive‐like characteristics with a diverse and private TCR repertoire in fetal tissue and clonal expansion in adults,^[^ ^22^, ^66^ ^]^ engage in stress surveillance. Additionally, we noted the expansion of NK and cDC1 subsets, which are also pivotal in immune surveillance,^[^ ^67^ ^]^ as well as an increase in γδ T ligand ANXA2 in GD TFCs. ANXA2 has been reported as an oxidative stress signal for Vδ2^−^γδ T cells.^[^ ^25^ ^]^ Furthermore, thyroid hormone synthesis generates reactive oxygen species (ROS), establishing a high‐ROS intracellular milieu.^[^ ^68^ ^]^ These clues collectively point to stress surveillance—where stressors (e.g., infection, psychological stress) exacerbate oxidative stress, overwhelming antioxidant defenses and damaging TFCs, while γδ T/NK cells detect TFC dysregulation and recruit immune effectors—as an important mechanism in GD pathogenesis.

It is noteworthy that MIF and MHC I/II were still the strongest molecules mediating the interaction between TFCs and immune components (Table S11, Supporting Information). Moreover, not all GD patients in our study display prominent γδ T subsets, suggesting that γδ T‐mediated stress surveillance may represent only one of multiple adaptive immune triggers. Future studies correlating γδ T frequency with genetic/environmental risks, disease severity, and treatment outcomes in larger cohorts will further enhance our understanding of the role of stress surveillance in GD.

Notably, γδ T in GD also highly express NK receptors (NKG2D, NKG2C) and share transcriptional profiles with trNK/CD56^bright^ NK cells. NKG2D recognizes stress‐induced ligands like MICA/B,^[^ ^42^, ^43^ ^]^ while NKG2C is associated with adaptive‐like NK responses during CMV infection.^[^ ^69^ ^]^ In addition to ANXA2, CD1D is also upregulated in thyroid tissue. CD1D was reported to activate γδ T/NK via endogenous phospholipid binding during viral‐induced cellular stress.^[^ ^70^ ^]^ Elevated levels of CD1D, CD1C, and HLA‐E were found in B cells in the GD group, though their roles in γδ T activation require validation. These results imply a wider involvement of molecular pathways and cell types in stress surveillance during GD development, extending beyond the ANXA2‐γδ T axis.

## 4 Conclusion

**Figure 8:**
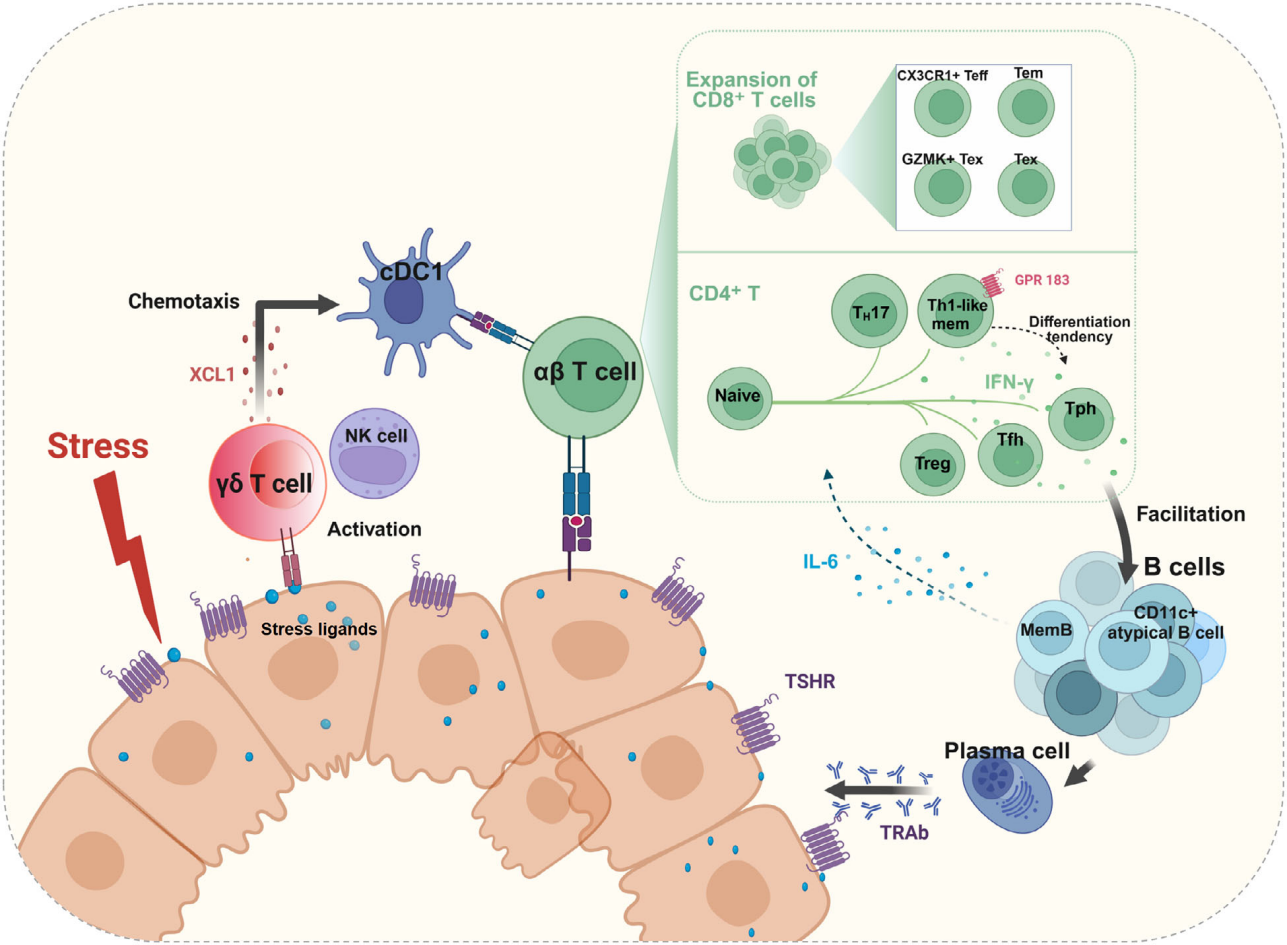
Summary schematic of the intrathyroidal immune microenvironment in GD. In GD thyroid tissue, CD4⁺ T cells exhibit a Th1‐like phenotype characterized by multiple IFN‐γ‐producing subsets and B helper features, accompanied by a notable expansion of CD8⁺ T subsets with effector properties. Tph cells and CD11c^+^ atypical B cells engage in extrafollicular B cell responses, while memory B cells modulate CD4^+^ T cell pattern formation through IL‐6. Beyond the conventional MHC‐dependent pathway, wherein antigen‐presenting cells (APCs) activate T cells to drive B cell differentiation into TSHR autoantibody‐producing plasma cells, our data suggest a complementary stress‐responsive mechanism. Specifically, TFCs in GD exhibited a stressed phenotype and elevated expression of stress‐induced self‐ligands. This microenvironment promotes the activation and expansion of stress‐surveilling γδ T/NK cells. These innate lymphocytes express XCL1/XCL2, which may facilitate the recruitment of cDC1 cells, leading to adaptive immune reorganization and suggesting a complementary, MHC‐independent pathway in GD pathogenesis. The diagram was drawn using Biorender.

Our study delineates the cellular architecture of GD thyroid tissue through single‐cell profiling (**Figure** **8**), revealing an IFN‐γ‐dominant CD4⁺ T cell milieu alongside expanded Tph cells that facilitate pathogenic extrafollicular B cell activation, as well as a notable increase in cytotoxic CD8⁺ T cells with effector properties. Furthermore, our data suggest a potential stress‐responsive axis in which stressed TFCs may activate γδ T/NK cells, which in turn recruit cDC1 via XCL1/XCL2 and contribute to immune reorganization. Collectively, these findings illuminate both adaptive and innate cellular mechanisms that may cooperate in GD pathogenesis.

## 5 Experimental Section

### Ethical Statement

This study was reviewed and approved by the Ruijin Hospital Ethics Committee, Shanghai Jiaotong University School of Medicine.

### Clinical Sample Collection

Thyroid tissue samples were obtained from thyroid disease patients undergoing thyroidectomy at Ruijin Hospital between 2018 and 2024, with complete thyroid function parameters and clinical history data. The clinical diagnosis of GD was confirmed by an endocrinologist according to Endocrine Society guidelines: history of overt hyperthyroidism; persistent TRAb positivity. Histological diagnosis of GD was validated postoperatively via hematoxylin and eosin (HE)‐stained sections reviewed by senior pathologists (Figure S10, Supporting Information). At the time of thyroidectomy, measurable TRAb concentrations were documented in all enrolled patients, with minimum titers of 10 IU L^−1^ (normal range <1.75 IU L^−1^). Control tissues were obtained from individuals undergoing surgery for non‐metastatic thyroid nodules or tumors. Strict exclusion criteria for AITD included: (1) normal reference‐range thyroid function (TSH 0.35–4.94 µIU mL^−1^, FT3 2.43–6.01 pmol L^−1^, FT4 9.01–19.04 pmol L^−1^); (2) undetectable TPOAb (<5.61 IU mL^−1^) and TGAb (<4.11 IU mL^−1^) by chemiluminescent assay. Sampling was performed >2 cm away from lesions, and histological normality was confirmed by HE/pathology review.

### Single‐Cell Isolation

Fresh surgical tissue specimens were dissociated into ≈1 mm^3^ fragments and subjected to enzymatic digestion with a cocktail of collagenase II (2 mg mL^−1^), collagenase IV (2 mg mL^−1^), and DNase I (0.1 mg mL^−1^). Digestion proceeded under continuous agitation at 37 °C for 30 min. The resulting suspension was filtered through a 40 µm nylon mesh, followed by two phosphate‐buffered saline (PBS) washes to remove debris. Cell viability and concentration were determined via AOPI‐based fluorescent staining. Processed cells were immediately prepared for downstream single‐cell genomic analysis using the 10x Genomics platform, adhering to manufacturer‐recommended workflows. Due to sample availability, processing was spread across multiple batches: each GD sample was processed individually, while control samples were batched as follows: (P02N, P04N), (P08, P11, P12), (P15, P16), with P29, P51, and P54 processed individually. Despite these logistical batch differences, all samples were processed using consistent protocols to minimize technical variation.

### scRNA‐seq and V(D)J Sequencing

Single‐cell libraries were prepared using the Chromium Next GEM Single Cell 5′ Kit v2 (10x Genomics) as per manufacturer guidelines. Cell suspensions combined with barcoded gel beads and partitioning oil were processed on a Chromium Chip to generate Gel Beads‐in‐Emulsion (GEMs). Intracellular RNA was barcoded via in‐GEM reverse transcription after cell lysis, followed by cDNA amplification. Transcriptome libraries were constructed using the 5′ Library Kit, while TCR libraries were enriched via V(D)J Kits. Sequencing was performed on an Illumina NovaSeq Xplus platform (2 × 150 bp paired‐end reads). The mean sequencing depth was 65, 167 read pairs per cell for scRNA‐seq and 35,381 read pairs per cell for scTCR profiling. Data processing utilized the 10x Genomics Cell Ranger pipeline (v3.0.1) for demultiplexing, barcode/UMI extraction, alignment to GRCh38, and generation of gene‐cell count matrices.

### Quality Control, Dimensionality Reduction and Clustering of scRNA‐seq Data

The raw expression matrix of each sample was converted into a Seurat object using the R package Seurat (version 4.3.0). Genes expressed in fewer than three cells were first excluded. Cells with fewer than 500 or more than 8000 unique feature counts, as well as those with unique molecular identifiers (UMIs) above the 98th percentile, were removed to exclude low‐quality cells. Doublets were excluded by using DoubletFinder (version 2.0.3),^[^ ^71^ ^]^ with an anticipated doublet rate of 7.5% (suitable for datasets with ≈10 000 recovered cells; the target was 8000). Using the “PercentageFeatureSet” function, cells with >15% mitochondrial genes, >40% ribosomal genes, or >3% hemoglobin genes were also discarded. Subsequently, these specific gene sets were excluded from the expression matrix. Following these steps, all samples were integrated and obtained a final dataset comprising 136 596 cells × 30 757 genes for downstream analysis.

Expression data were normalized for each cell using the “NormalizeData” function with default parameters. The top 2000 highly variable genes (HVGs) identified by the “FindVariableFeatures” function were utilized as input for principal component analysis (PCA) performed with the “RunPCA” function. To address batch effects, Harmony integration was applied using the “RunHarmony” function. The top 30 principal components were subsequently used for cell clustering, performed with the “FindNeighbors” and “FindClusters ”functions. Finally, the “RunUMAP” function was utilized for dimensionality reduction and visualization.

To conduct subcluster analysis within each major cell cluster, cells of interest were isolated using the ‘subset’ function. Clustering and dimensionality reduction were repeated using UMAP as previously described. The top principal components were selected based on the results of the “ElbowPlot” function.

### Functional Annotation of Cell Clusters

To identify and annotate cell clusters, the FindAllMarkers function was used in Seurat, with statistical significance assessed using Bonferroni correction. Marker genes for each cluster were defined as those exhibiting a Bonferroni‐adjusted *p*‐value < 0.05, a log_2_ fold change (log_2_FC) > 0.25, and expression in more than 25% of cells in at least one of the two compared clusters. Each cluster was subsequently annotated based on the expression of canonical marker genes (Table S2, Supporting Information).

To validate these annotations, SingleR (version 1.0.1) was employed, a reference‐based supervised method for automated cell type identification. Five human reference datasets were used: MonacoImmuneData, DatabaseImmuneCellExpressionData, BlueprintEncodeData, HumanPrimaryCellAtlasData, and NovershternHematopoieticData, along with a combined reference integrating all five. SingleR assigns the most probable cell type label to each query cell by calculating the correlation between its gene expression profile and those of reference cell types. The results were then aggregated at the subpopulation level to generate final cell type annotations (Table S12, Supporting Information).

### Cell Proportion Analysis

Differential abundance analysis between GD and control samples was performed using the miloR package (v2.5.1).^[^ ^72^ ^]^ A k‐nearest neighbor (KNN) graph was first constructed from a SingleCellExperiment object (buildGraph), which was then used to define cellular neighborhoods (makeNhoods). Differential abundance was tested within each neighborhood (testNhoods), with the false discovery rate (FDR) corrected for spatial topology (calcNhoodDistance). Subsequently, neighborhoods were annotated with a dominant cell type if it constituted ≥70% of the cells (annotateNhoods); otherwise, they were labeled as “Mixed” and excluded from visualization. Neighborhoods with a spatially corrected FDR < 0.05 were considered significant.

### Gene Expression and Pathway Enrichment Analysis

Differential gene expression analysis across clusters was performed using the function FindMarkers, genes expressed in more than 10% of cells within a cluster, along with a FC > 2 (or >1.5) compared to the other clusters, were identified as DEGs and used for pathway enrichment analysis.

Pathway enrichment analysis was performed using the R package clusterProfiler (v4.13.0). Significantly enriched terms were identified based on an adjusted *p*‐value < 0.05. Gene Ontology (GO) analysis was performed using the enrichGO function for the differentially expressed genes (DEG) of the following comparisons: Th1 cells versus other T cells, γδ T cells versus other T cells, among NK cell subsets, TFCs in GD versus control, and the M1 mural cell subset in GD versus control. For the NK cell subsets, KEGG analysis was also conducted using the enrichKEGG function. To better visualize enriched biological themes, Gene Set Enrichment Analysis (GSEA) was applied via the gseGO function for TFCs and γδ T cell comparisons.

### TCR Data Analysis

Using the human GRCh38 genome as the reference, the 10× Genomics Cell Ranger V(D)J pipeline was employed to identify TCR clonotypes through alignment and annotation with the default parameters. Clonotype information for each cell was manually added as cell metadata to the Seurat object, and this annotation was used to explore the relationship between cell types and TCR sequences. Cells sharing identical CDR3α and CDR3β pairs were defined as a clonotype. If a clonotype was present in at least two cells, the cells carrying this clonotype were defined as clonal cells.

The degree of clonal expansion within each subtype was defined as the ratio of the total number of cells sharing the same clonotypes to the total number of cells with TCR information in that subtype. In each sample, the expansion value was calculated as below:

(1) $$\mathrm{expansion}\_\mathrm{value}\;=\frac{\sum_{i=1}^{k}n_{i}}{N}$$

k: number of clonal cells, n_i_: number of cells for clonotype i, N: total number of cells in a subtype

The mean expansion value for each subtype in the GD and control samples was calculated separately and used to generate a heatmap.

The degree of clonal transition between two subtypes was calculated as the ratio of the total number of cells sharing the same clonotype to the total number of cells with TCR information across the two subtypes. In each sample, the transition value between each pair of subtypes was calculated as below:

(2) $$\mathrm{transition}\_\mathrm{value}\;=\frac{\sum_{i=1}^{k}n_{i-\mathrm{subtypeA}}+n_{i-\mathrm{subtypeB}}}{N}$$

k: number of clonal cells present in both subtypes A and B, n_i‐subtypeA_: number of cells for clonotype i in subtypeA, n_i‐subtypeB_: number of cells for clonotype i in subtypeB, N: total number of cells in subtypes A and B.

The mean transition value between each pair of subtypes in the GD and control samples was calculated separately and used to generate the network in Cytoscape (version 3.10.2).

### CytoTRACE Analyses

Differentiation potential was assessed using CytoTRACE2 (version 1.0.0),^[^ ^73^ ^]^ an interpretable deep learning framework designed to characterize cellular potency. The resulting CytoTRACE scores offer a continuous measure of developmental potential, ranging from 0 (more differentiated) to 1 (less differentiated), derived from normalized absolute predicted potency scores.

### Trajectory Analysis

Cell‐state transitions within CD4+ and CD8+ T cell subtypes were independently inferred using Monocle2 (v2.24.0).^[^ ^74^ ^]^ A NewCellDataSet object was constructed from the counts matrix using the parameter: lowerDetectionLimit = 0.5, expressionFamily = negbinomial.size(). Size factors and dispersions were estimated with default settings. For trajectory construction, genes were selected based on mean expression ≥ 0.1 and empirical dispersion ≥ 1 × fitted dispersion, as identified by “dispersionTable” function. Dimensionality reduction was performed via DDRTree. The cell with the highest CytoTRACE score was designated as the root for orderCells. Pseudotime‐associated and branch‐dependent differentially expressed genes (DEGs) were identified using “differentialGeneTest” and “BEAM” function, respectively. Trajectories were visualized with “plot_cell_trajectory” function and colored by metadata (e.g., subtype, pseudotime).

Trajectory robustness was validated using Monocle3 (v1.3.7) and Cellrank2 (v2.0.7).^[^ ^75^, ^76^ ^]^ In Monocle3, the trajectory was constructed with learn_graph (learn_graph_control = list(minimal_branch_len = 20, geodesic_distance_ratio = 0.5)), using the highest CytoTRACE‐scoring cell as the root. In Cellrank2 (Python v3.9.23), a transition matrix was computed using a PseudotimeKernel with CytoTRACE scores as the “time_key” and a soft threshold scheme.

### Cell–Cell Communication Analysis

Cell–cell communication analysis and visualization were performed using the R package CellChat (version 2.1.2), focusing on the expression of known ligand‐receptor pairs between cell clusters. In addition, the “updateCellChatDB” function was employed to incorporate ligand‐receptor pairs specific to γδ T cells into the database to enhance the detection of γδ T cell‐mediated intercellular interactions. Using the previously integrated scRNA‐seq data, CellChat objects were generated separately for GD and control samples via the “createCellChat” function, followed by cell type annotation and identification of overexpressed genes. Signaling pathway networks among all cell subclusters were subsequently analyzed. Communication probabilities were inferred using the “computeCommunProb” function, with the trimean method applied to calculate the average gene expression per cell group, resulting in stronger interactions.

### RNA Extraction and qRT‐PCR

Total RNA was extracted from liquid nitrogen‐snap‐frozen surgical thyroid samples using Trizol as standard protocols, cDNA was reverse‐transcribed using the Reverse Transcription Kit (Vazyme) according to the manufacturer's protocol. qRT‐PCR was performed in triplicates in a 384‐well plate using SYBR Green Master Mix (Vazyme) with primers listed in Table S13 (Supporting Information).

### Flow Cytometry

Cells were stained with Zombie Aqua Fixable Viability Kit (BioLegend) to exclude dead cells, blocked with Human TruStain FcX (BioLegend). Antibodies used for cell staining include: CD3‐PerCP/Cy5.5(BioLegend, #317336, 1:100), CD4‐PE/Cy7(BioLegend, #317414, 1:100), IFN‐γ‐BV605 (BioLegend, #506542, 1:100), IL‐17A‐PE (BioLegend, #512306, 1:100), IL‐4‐APC (BioLegend, #500812; 1:100), FOXP3‐BV421 (BioLegend, #320124; 1:100), and TCR γ/δ‐BV421 (BioLegend, #331217; 1:100). For intracellular targets, cells were stimulated with PMA (50 ng mL^−1^; Yeasen), ionomycin (500 ng mL^−1^; Yeasen), and brefeldin A (5µg mL^−1^; Yeasen) for 5 hSamples were analyzed on a CytoFLEX flow cytometer (Beckman Coulter) with FlowJo 10.0.

### Immunofluorescent Staining

Formalin‐Fixed Paraffin‐Embedded sections of thyroid tissues were deparaffinized, rehydrated, and antigen‐retrieved in Tris‐EDTA (pH 9.0, 100 °C, 20 min). Endogenous peroxidase was blocked with 3% H_2_O_2_, followed by DAKO blocking buffer. Primary antibodies, anti‐CD3 (Cell Signaling Technology, #85061, 1:300) and TCR γ/δ (Santa Cruz Biotechnology, #sc‐100289, 1:100) were incubated overnight at 4 °C. After PBS washes, sections were stained with Donkey anti‐Mouse IgG H&L‐Alexa Fluor 594 (Yeasen, #34112ES60, 1:500), Donkey anti‐Rabbit IgG H&L ‐Alexa Fluor 488 (Yeasen, #34206ES60, 1:500). DAPI Fluoromount‐G (SouthernBiotech, #0100‐20) was used for nuclear staining. Images were acquired on a Zeiss LSM880 confocal microscope.

### Multicolor Immunohistochemistry

The antibodies used in this section were: anti‐CD4 (Abcam, clone SP7, diluted at 1:400), anti‐CD11c (Abcam, clone EPR6855, diluted at 1:400), anti‐TCR δ (Abcam, clone 144B, diluted at 1:500) and anti‐XCR1 (Abcam, clone mAbcam22510, diluted at 1:500). The antigenic binding sites were visualized using the Opal 7‐Color Manual IHC Kit (PerkinElmer, NEL811001KT) according to the protocol of the manufacturer.

Multicolor immunohistochemistry data were collected by Mantra Quantitative Pathology Workstation (PerkinElmer, CLS140089) and analyzed by InForm 2.2.1.

### Histological Staining

FFPE sections were deparaffinized, rehydrated, and treated with 3% H_2_O_2_. Antigen retrieval used Tris‐EDTA (pH 9.0). Sections were permeabilized with 0.2% Triton X‐100, blocked, incubated with primary antibodies anti‐Annexin A2 (Abcam, #ab41803, 1:1000), anti‐Granzyme K (Abcam, #ab282703) or anti‐TCR δ (Santa Cruz, #sc‐100289) at 4 °C overnight, then HRP‐conjugated goat anti‐mouse/rabbit secondary antibody (RecordBio, #RCB054). DAB (Servicebio, #G1212) staining, hematoxylin counterstaining, dehydration, and mounting followed. Imaging via TissueFAXS Plus S slide scanner (TissueGnostics); quantification with QuPath.

### Primary Thyroid Cell Isolation and Culture

Fresh thyroid tissues from GD or control donors were washed three times with PBS containing 1% penicillin–streptomycin and minced into small fragments. The tissue pieces were transferred into gentleMACS C Tubes (Miltenyi Biotec, #130‐093‐237) and digested in Advanced DMEM/F12 (Gibco, #12634‐010) supplemented with 1×GlutaMAX (Gibco, #35050‐061), 10 µM Y‐27632 (MCE, #HY‐10583), 100 µg mL^−1^ Primocin (InvivoGen, #ant‐pm‐05), and 1 mg mL^−1^ collagenase (Sigma–Aldrich, #C9407) at 37 °C for 30 min using a gentleMACS Dissociator (Miltenyi Biotec, #130‐093‐235). The digestion was terminated by adding complete medium containing 10% FBS, and then passed through a 70‐µm cell strainer, centrifuged at 500 × g for 8 min at 4 °C, and subjected to red blood cell lysis. The resulting cell pellet was resuspended in complete culture medium consisting of Advanced DMEM/F12 supplemented with 10% FBS, 1× ITS, 1× penicillin–streptomycin, and 1× GlutaMAX, and subsequently seeded into culture plates for further experiments.

### Co‐Culture of Primary TFCs with γδ T Cells

Primary human Vδ1⁺ γδ T cells, isolated from peripheral blood mononuclear cells (PBMCs), were a gift from Prof. Lin Yang at Cyrus Tang Medical Institute, Soochow University, China. These cells were cultured according to the provider's established protocol.^[^ ^77^, ^78^ ^]^ Briefly, Vδ1⁺ γδ T cells were maintained in X‐VIVO 15 medium (Lonza, 04–418Q) supplemented with 100 IU mL^−1^ human IL‐2 (Novoprotein, GMP.CD66), 20 ng mL^−1^ human IL‐15 (Novoprotein, GMP.C016), and 30 ng mL^−1^ human IL‐21 (Novoprotein, GMP.CC45), and CD3/CD28 Dynabeads (Gibco, 11131D) at a 1:1 bead‐to‐cell ratio. After 7 days of culture, the cells were used for subsequent co‐culture experiments.

Primary TFCs were adapted to the γδ T cell medium for 24 h prior to co‐culture. To establish a stressed TFC model, primary TFCs were treated with 60 µm H_2_O_2_ (Sigma–Aldrich, #88597) for 30 min. Subsequently, γδ T cells were added to the TFCs at a density of 2 × 10⁶ cells mL^−1^, with 3:1 TFCs‐to‐γδ T cells ratio.

For the activation assay, γδ T cells were harvested after 48 h of incubation. Vδ1⁺ γδ T cell activation was then evaluated by flow cytometry (CytoFLEX, Beckman Coulter) based on the surface expression of CD69 (BV605‐conjugated anti‐CD69, BD Biosciences, #562989, 1:100) and CD25 (PerCP‐Cyanine5.5‐conjugated anti‐CD25, eBioscience, #45‐0251‐82, 1:100).

For the proliferation assay, Vδ1⁺ γδ T cells were first labeled with CFSE (BD Biosciences, #565082) at the end of the 7‐day pre‐culture period. After 60 h of co‐culture with TFCs, γδ T cells were harvested to assess proliferation. Proliferation was quantified by measuring CFSE fluorescence dilution in Vδ1⁺ γδ T cells, which were gated as CD3⁺ (APC/Cyanine7‐conjugated anti‐CD3, Invitrogen, #MA5‐38705, 1:100) to identify the T‐cell population.

### ROS Assay

NTHY cells were seeded in 6‐well plates at a density of 2.5 × 10⁶ cells per well and cultured under standard conditions (37 °C, 5% CO_2_) until they reached ≈80% confluence. The cells were then treated with different concentrations (0, 100 ng mL^−1^, 500 ng mL^−1^, and 10 µg mL^−1^) of LPS (InvivoGen, #tlrl‐eblps) for 12 h or 100 µm H_2_O_2_ for 30 mins. After treatment, the cells were incubated with 10 µm H_2_DCFDA (MedChemExpress, #D0940) for 30 min, and then Hoechst (Beyotime, #C1029) to visualize nuclei. ROS levels were subsequently analyzed using flow cytometry (Beckman coulter) and fluorescence microscopy (Nexcope).

### ANXA2 Level Analysis in Iodine Diet Mice

The FFPE sections of thyroid tissues from high iodine intake NOD.H‐2^h4^ mice were kindly provided by Professor Yushu Li and Na Zhao in the First Affiliated Hospital, China Medical University. NOD.H‐2^h4^ mice at 4 weeks of age received drinking water supplemented with 0.05% NaI or normal drinking water, whereafter they were sacrificed at 12 weeks of age for the collection of thyroid tissue. Immunohistochemical staining was performed as described above.

### Gene‐Set Analysis of GWAS Data

A genome‐wide association study (GWAS)‐based gene‐set enrichment analysis was conducted using MAGMA (version 1.10), based on the previously reported 28 genomic risk loci associated with GD. The European reference panel from phase 3 of the 1000 genomes project was used as the reference population. Marker genes with a fold change (FC) > 1.5 in each subcluster were selected as the gene sets. These gene sets were used to test which subclusters were associated with genetic traits at the gene level.

### Statistical Analysis

All the statistical analyses were performed using R (version 4.0.5). Statistical details, including *n* value, number of experiments, *P* values, and the type of statistical tests used for each experiment, can be found in the figure legends. *p* < 0.05 was considered statistically significant. Additional visualization (e.g., Violin plots, Box plots, UMAPs, and heatmaps) was performed using the R package ggplot2 (version 3.5.0), ggpubr (version 0.6.1), pheatmap (version 1.0.13), and Seurat(version 4.3.0).

## Conflict of Interest

The authors declare no conflict of interest.

## Author Contributions

X.Z., J.C., R.P., and D.Y. are co‐first authors and contributed equally to this work. X.Z. and J.C. performed data analysis. R.P., D.Y., and C.D. performed molecular experiments. X.Y. and L.S. also contributed to experiments. R.P. and J.K. collected samples. J.X. performed a pathological diagnosis. J.Y. and Y.Z. contributed to the clinical data. F.L. and L.S.Y. contributed in the immune‐related analysis. H.Y. and R.H. contributed to data management. X.Z. and L.Y. wrote the manuscript. N.G., S.W., W.W., and L.Y. conceived the project and designed the studies.

## Supporting information



Supporting Information

Supporting Information

## Data Availability

The data that support the findings of this study are openly available in [Genome Sequence Archive in National Genomics Data Center at GSA‐Human] at [https://ngdc.cncb.ac.cn/search/specific?db=hra&q=HRA010993], accession number [HRA10993].
