# Supplementary material for: Single‐Cell RNA Sequencing of Thyroid Tissues Reveals Pathogenesis of Graves' Disease
Source: Adv Sci (Weinh). 2025 Oct 23;13(1):e08449. doi: 10.1002/advs.202508449 (PMC12767108; doi:10.1002/advs.202508449)
Supplement: Supplementary file 1 — Supporting Information [file ADVS-13-e08449-s001.pdf]

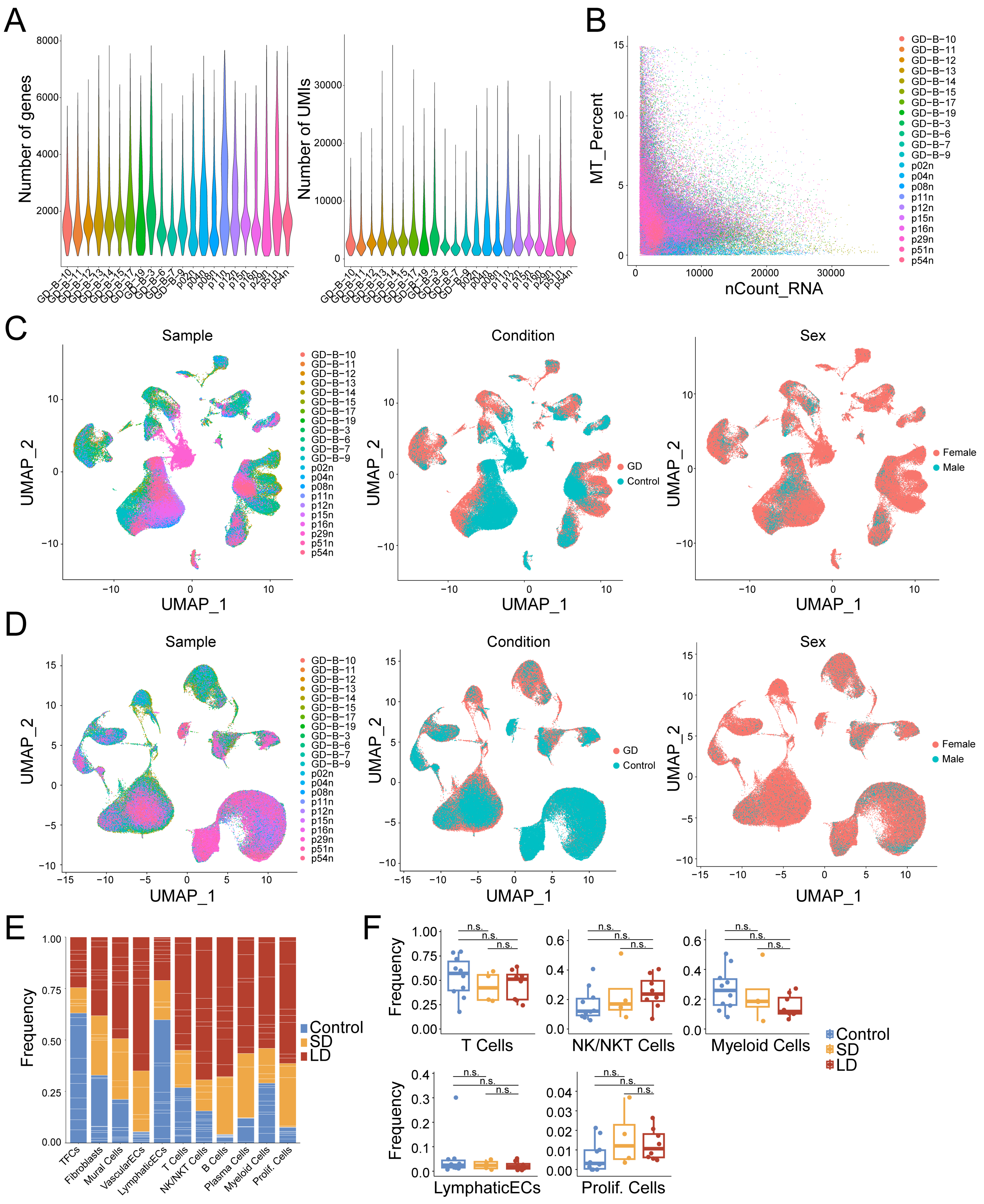

Figure S1. Basic information of the scRNA-seq data in samples. A) Number of genes and UMIs per cell detected in each sample after quality control. **B)** Dot plot showing the relationship between mitochondrial gene percentage and UMI counts per cell. **C-D)** UMAP plots of all cells (N = 136596) before **(C)** and after **(D)** batch correction using the Harmony algorithm. Cells are colored by patients, conditions, or sexes. **E)** Bar plots showing the distribution of 11 major cell groups across all samples. One block represents one individual sample. **F)** Proportion of each cell group (immune and non-immune cells calculated separately). Horizontal lines represent median values, and each dot signifies one sample. Wilcoxon rank-sum test.

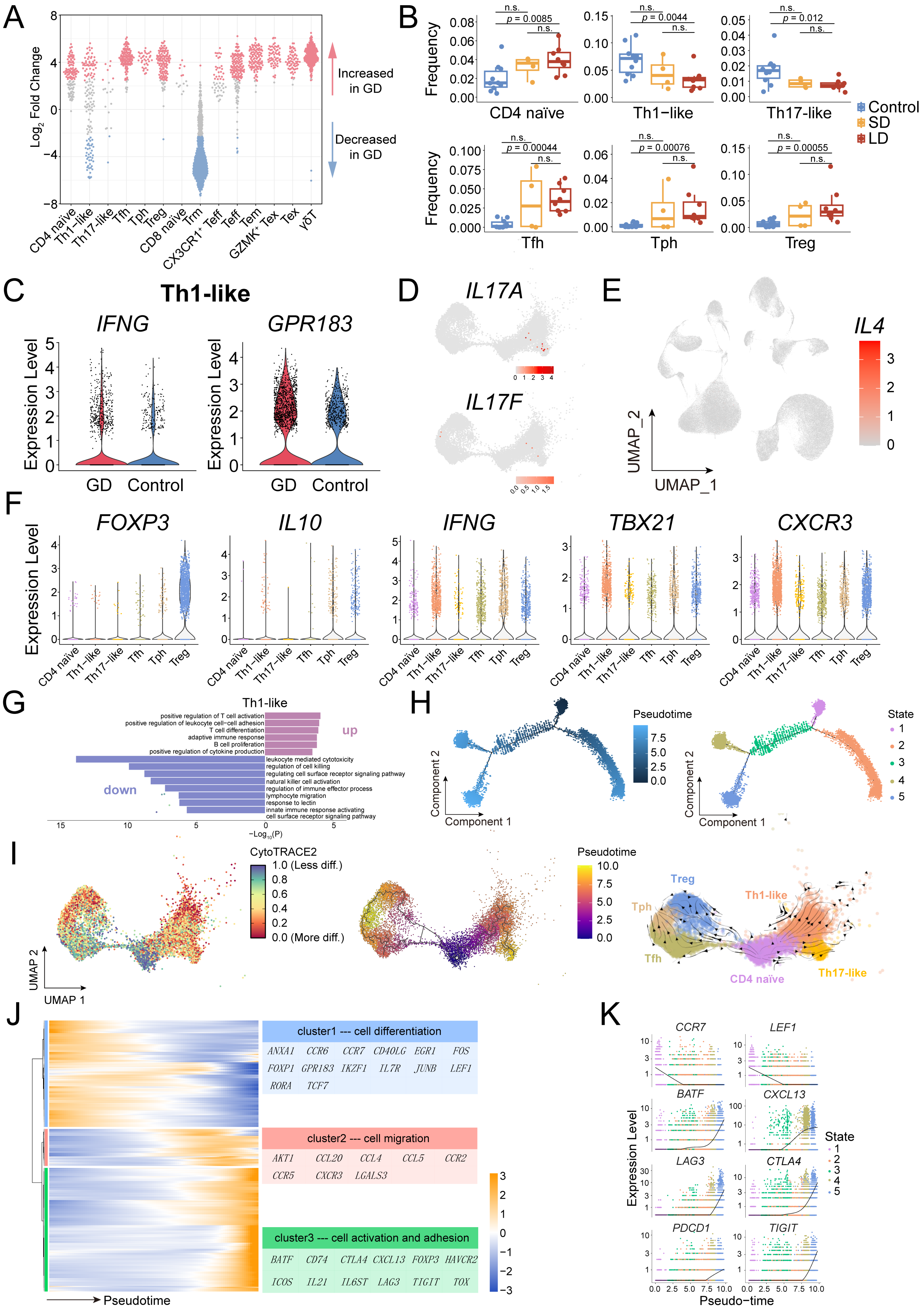

A
Log2 Fold Change
Increased in GD
Decreased in GD
CD4 naïve
Th1-like
Th17-like
Tfh
Tph
Treg
CD8 naïve
Trm
CX3CR1+ Teff
Teff
Tem
GZMK+ Tex
Tex
γδT
B
Frequency
CD4 naïve
Th1−like
Th17−like
Tfh
Tph
Treg
Control
SD
LD
n.s.
p = 0.0085
p = 0.0044
p = 0.012
p = 0.00044
p = 0.00076
p = 0.00055
C
Th1-like
IFNG
GPR183
Expression Level
GD
Control
D
IL17A
IL17F
E
IL4
UMAP_1
UMAP_2
F
FOXP3
IL10
IFNG
TBX21
CXCR3
Expression Level
G
Th1-like
positive regulation of T cell activation
positive regulation of leukocyte cell−cell adhesion
T cell differentiation
adaptive immune response
B cell proliferation
positive regulation of cytokine production
leukocyte mediated cytotoxicity
regulation of cell killing
regulating cell surface receptor signaling pathway
natural killer cell activation
regulation of immune effector process
lymphocyte migration
response to lectin
innate immune response activating
cell surface receptor signaling pathway
up
down
−Log10(P)
H
Pseudotime
State
Component 1
Component 2
I
CytoTRACE2
1.0 (Less diff.)
0.0 (More diff.)
Pseudotime
Treg
Th1-like
Tph
Tfh
CD4 naïve
Th17-like
UMAP 1
UMAP 2
J
cluster1 --- cell differentiation
ANXA1 CCR6 CCR7 CD40LG EGR1 FOS
FOXP1 GPR183 IKZF1 IL7R JUNB LEF1
RORA TCF7
cluster2 --- cell migration
AKT1 CCL20 CCL4 CCL5 CCR2
CCR5 CXCR3 LGALS3
cluster3 --- cell activation and adhesion
BATF CD74 CTLA4 CXCL13 FOXP3 HAVCR2
ICOS IL21 IL6ST LAG3 TIGIT TOX
Pseudotime
K
CCR7
LEF1
BATF
CXCL13
LAG3
CTLA4
PDCD1
TIGIT
Expression Level
State
Pseudo−time

Figure S2. Properties of CD4$^+$ T cell subsets, related to Fig.2. A) Beeswarm plots illustrate the enrichment (red) or decrease (blue) of neighborhoods in GD for T cell subtypes calculated using MiloR (FDR < 0.05). **B)** Box plots showing the proportion of six CD4$^+$ T cell subsets among immune cells. Wilcoxon rank-sum test. **C)** Violin plots showing elevated *IFNG* and *GPR183* expression in Th1-like memory subset of GD compared to control. **D)** The expression level of *IL17A* and *IL17F* CD4$^+$ T cell subsets. **E)** The expression level of *IL4* is very low in all 11 major cell groups. **F)** Violin plots showing representative gene expression across CD4$^+$ T cell subsets. **G)** Pathway enrichment analysis of DEGs between Th1-like subset and other T cells. **H)** Pseudotime analysis of CD4$^+$ T cell subsets using Molocle2, colored by pseudotime (left) or state (right). **I)** Pseudotime analysis of CD4$^+$ T cell subsets using CytoTRACE2 to assess differentiation potential (left); trajectory inference using Monocle3, with CytoTRACE scores used to identify the "root" (mid); trajectory inference using Cellrank2 with CytoTRACE scores as the "time key" (right). **J)** Heatmap revealing three clusters of genes (q < $1e^{-4}$) that co-vary across pseudotime. **K)** Dynamic expression of representative genes during the pseudotime progression.

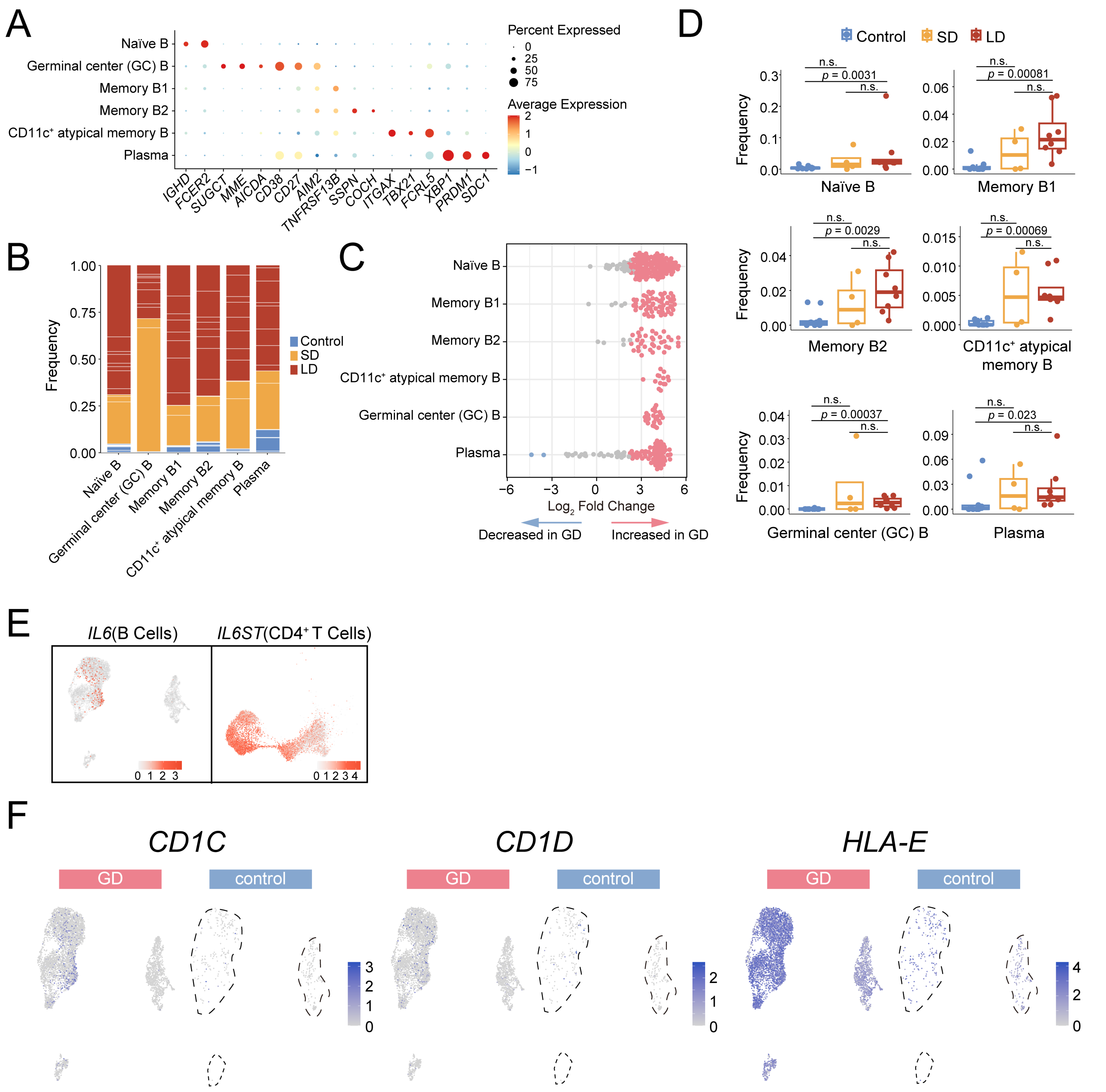

Figure S3. The identification and signatures of B cell subsets. A) Dot plot showing the expression of representative marker genes used for B cell subsets identification. **B)** Bar plots showing the distribution of six B cell subsets across all samples. Blocks represent individual samples. **C)** Beeswarm plots illustrate the enrichment (red) or decrease (blue) of neighborhoods in GD for B cell subtypes calculated using MiloR (FDR < 0.05). **D)** Box plots showing the proportion of six B cell subsets among immune cells. Wilcoxon rank-sum test. **E)** *IL-6* is primarily expressed in memory B1, while *IL6ST* is expressed in multiple CD4⁺ T cell subsets. **F)** Differential expression of γδ TCR ligand gene (*CD1C*, *CD1D*) and KLRC2 ligand gene (*HLA-E*) between GD and control groups.

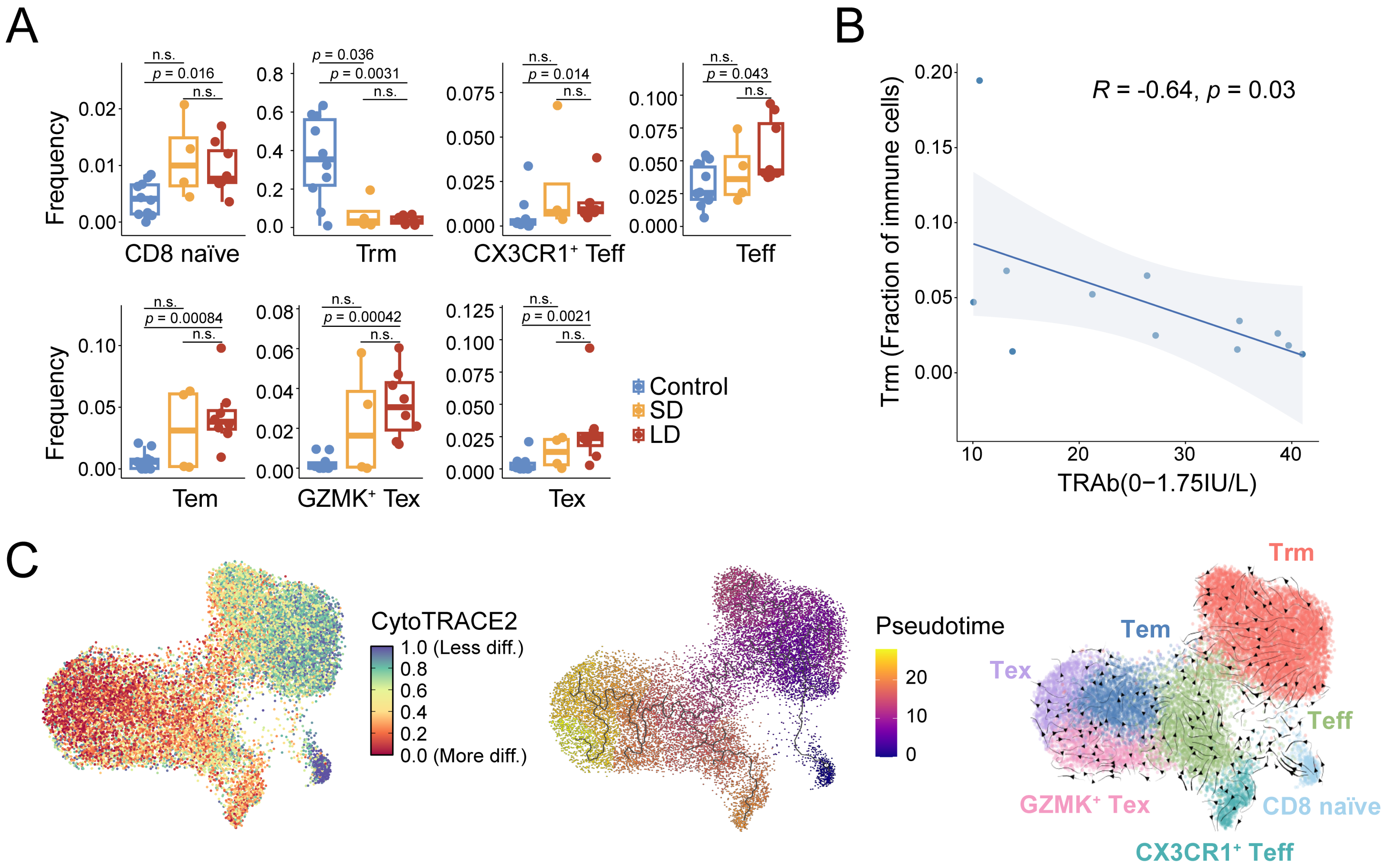

Figure S4. Proportions of seven CD8⁺ T cell subsets and association of Trm frequency with TRAb. A) Box plots showing the proportion of seven CD8⁺ T cell subsets among immune cells. Wilcoxon rank-sum test. **B)** The frequency of Trm subsets shows a negative association with TRAb levels in GD. Each dot represents one individual sample. Spearman's correlation test. **C)** Pseudotime analysis of CD8⁺ T cell subsets using CytoTRACE2 to assess differentiation potential (left); trajectory inference using Monocle3, with CytoTRACE scores used to identify the "root" (mid); trajectory inference using Cellrank2 with CytoTRACE scores as the "time key" (right).

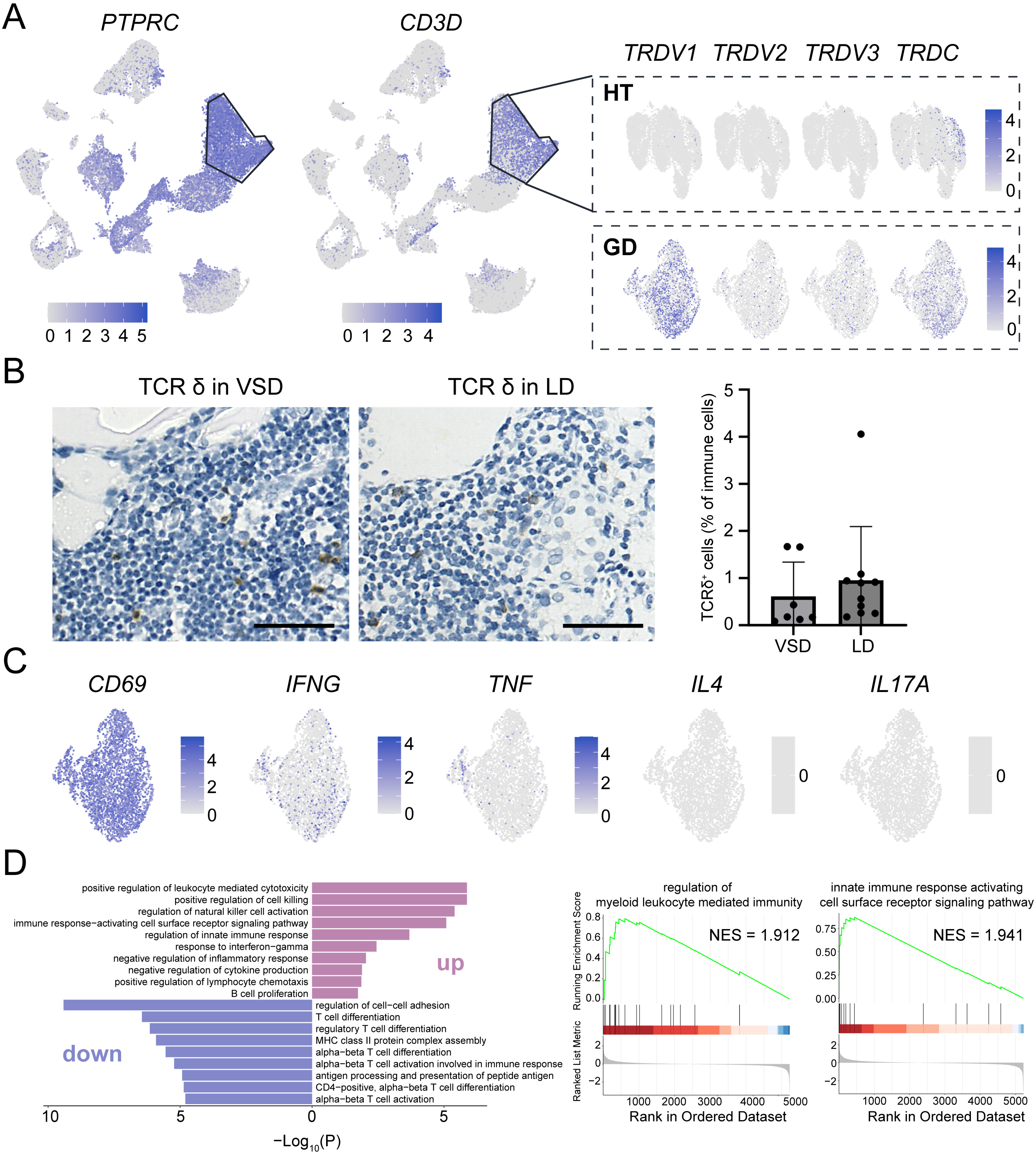

Figure S5. Characteristics of thyroid tissue γδ T cells, related to Fig. 3. A) TCR δ chain coding genes exhibit minimal expression in $CD45^{+}CD3^{+}$ T cells from HT, indicating the absence of substantial γδ T cell populations. In contrast, T cells in GD demonstrate predominant expression of *TRDV1* and *TRDC*. HT scRNA-seq data were derived from public dataset. [39] **B)** Representative TCRδ IHC staining in GD thyroid tissue. bar = 50 μm. Quantification (right) revealed no significant difference (p = 0.498) in the proportion of $TCR\delta^{+}$ cells among total immune cells between the very short duration (VSD, < 2 months, n = 7) and long duration (LD, 84-240 months, n = 10) groups. Data are presented as mean ± s.d., two-tailed Student's t-test. **C)** UMAP plots displaying expression of representative genes in γδ T cells (N = 3484). **D)** Pathway enrichment analysis of DEGs between γδ T and other T cells revealed distinct functions in γδ T cells.

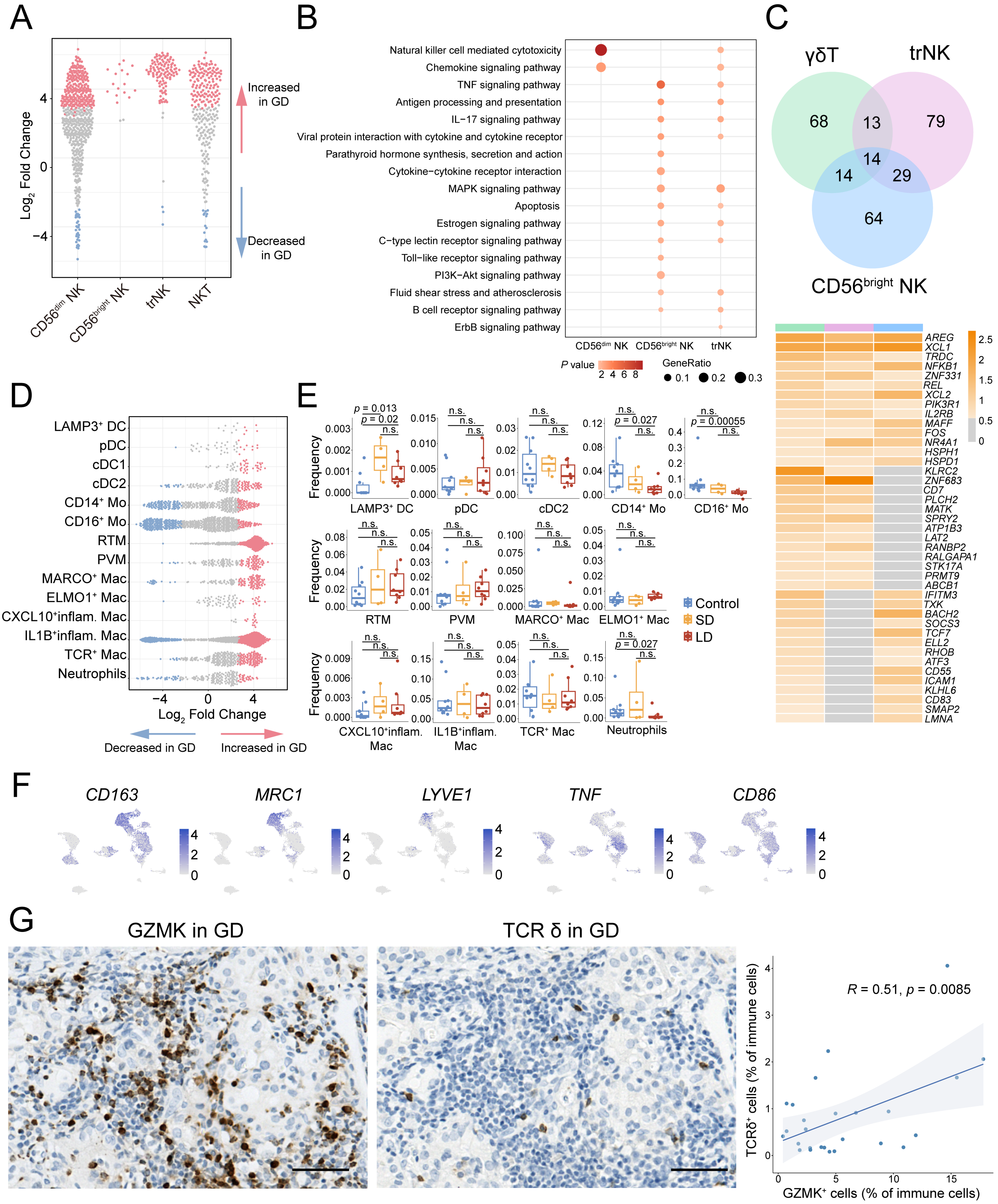

Figure S6. Characteristics of NK and myeloid cells, related to Fig. 4. A) Beeswarm plots illustrate the enrichment (red) or decrease (blue) of neighborhoods in GD for NK/NKT cell subsets calculated using MiloR (FDR < 0.05). **B)** KEGG enrichment analysis of DEGs in the three NK cell subsets revealed their distinct functions. **C)** Representative shared genes across trNK, CD56^{bright} NK and γδ T cells were visualized by Venn diagram (upper) and heatmap (lower). **D)** Beeswarm plots illustrate the enrichment (red) or decrease (blue) of neighborhoods in GD for myeloid cell subtypes calculated using MiloR (FDR < 0.05). **E)** Proportions of myeloid cell subsets among immune cells in different groups. Wilcoxon rank-sum test. **F)** UMAP plots displaying expression of representative genes in myeloid cells. **G)** Representative IHC staining of GZMK and TCRδ in lymphocytic infiltrates of GD thyroid tissue (left), and correlation between their positive cell proportions (right). bar = 50 µm. Pearson's correlation test.

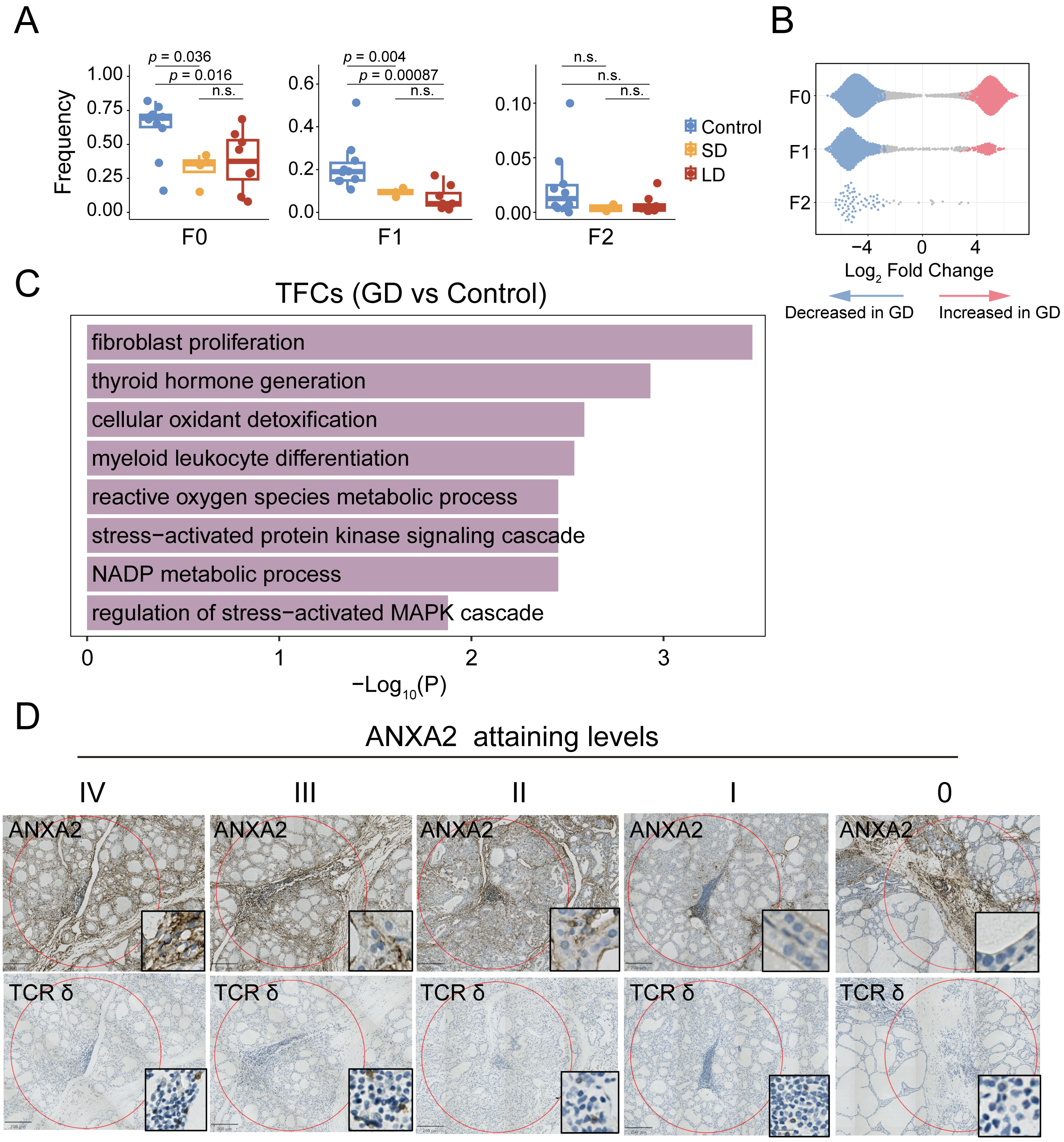

Figure S7. Characteristics of TFC subsets, related to Fig.5. A) Box plots showing the proportions of three TFC subsets among non-immune cells. Wilcoxon rank-sum test. **B)** Beeswarm plots illustrate the enrichment (red) or decrease (blue) of neighborhoods in GD for TFC subtypes calculated using MiloR (FDR < 0.05). **C)** Bar plots displaying enriched GO terms in DEGs of TFC in GD compared to the control group. **D)** Correlation scheme for ANXA2 expression and γδ T cell proportions in GD thyroid FFPE sections: Lymphocytic infiltrates were identified by HE staining, and then a 1 mm^2 area (red circle) was delineated centered on this region. ANXA2 in TFCs was graded (0/I-IV). The proportion of γδT cells was calculated by QuPath software as the ratio of TCRδ positive cells to the total number of lymphocytes within the region. bar = 200μm.

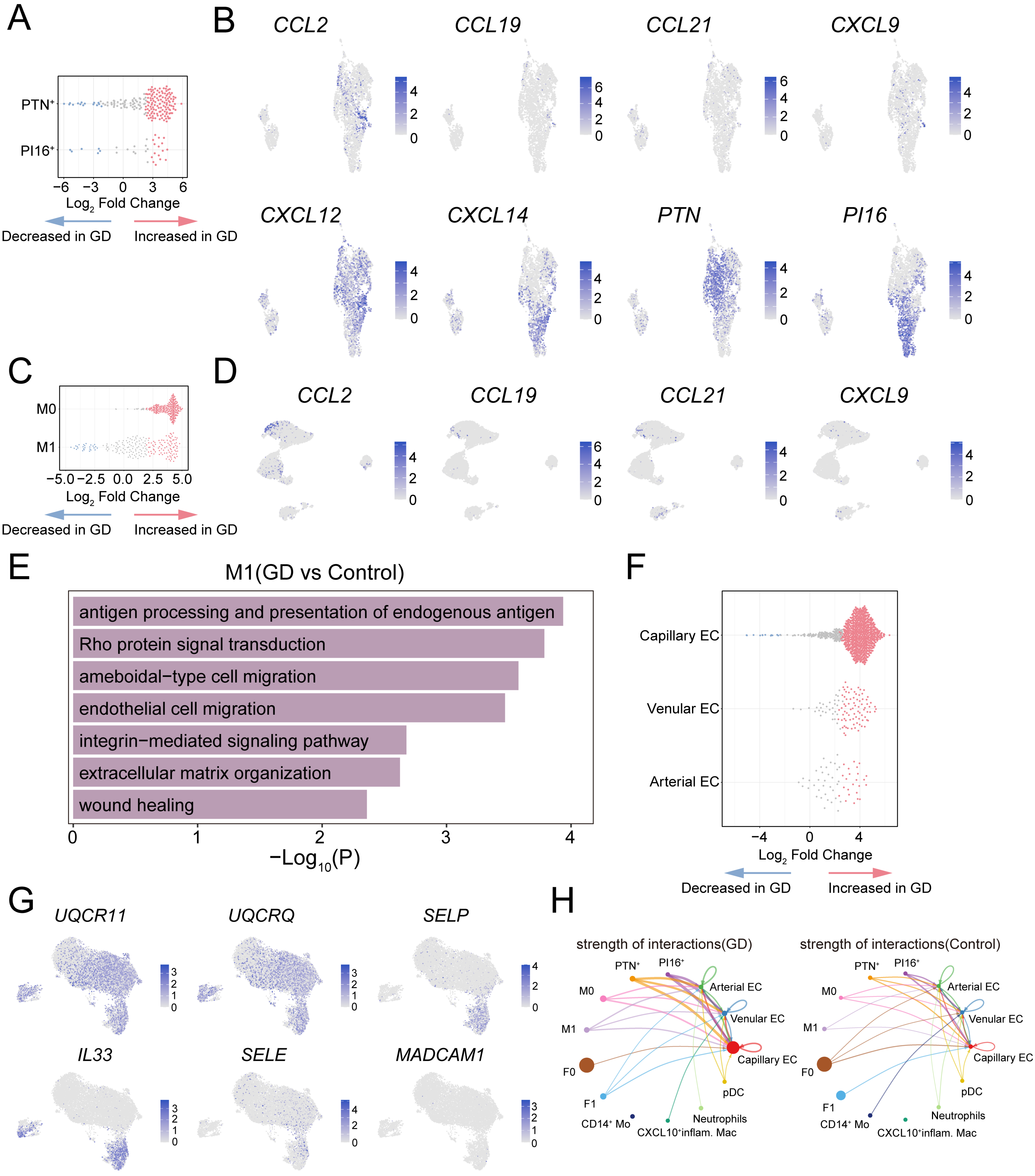

Figure S8. Characteristics of thyroid stromal cell subsets, related to Fig.7. A, C, F) Beeswarm plots illustrate the enrichment (red) or decrease (blue) of neighborhoods in GD for fibroblast subsets **(A)**, mural subsets **(C)**, and vascularEC subsets **(F)**, calculated using MiloR (FDR < 0.05). **B)** UMAP plots displaying expression of fibroblast marker genes and specific chemokines in fibroblast subsets (N = 3718). **D)** UMAP plots displaying expression of specific chemokines in mural cells (N = 7542). **E)** Bar plots displaying enriched GO terms in DEGs of M1 subsets comparing GD with the control group. **G)** UMAP plots displaying expression of selected marker genes in vascular endothelial cells (N = 13909). **H)** Circle plot representing the strengthening of three vascular endothelial cell subsets as receivers and their top 10 senders.

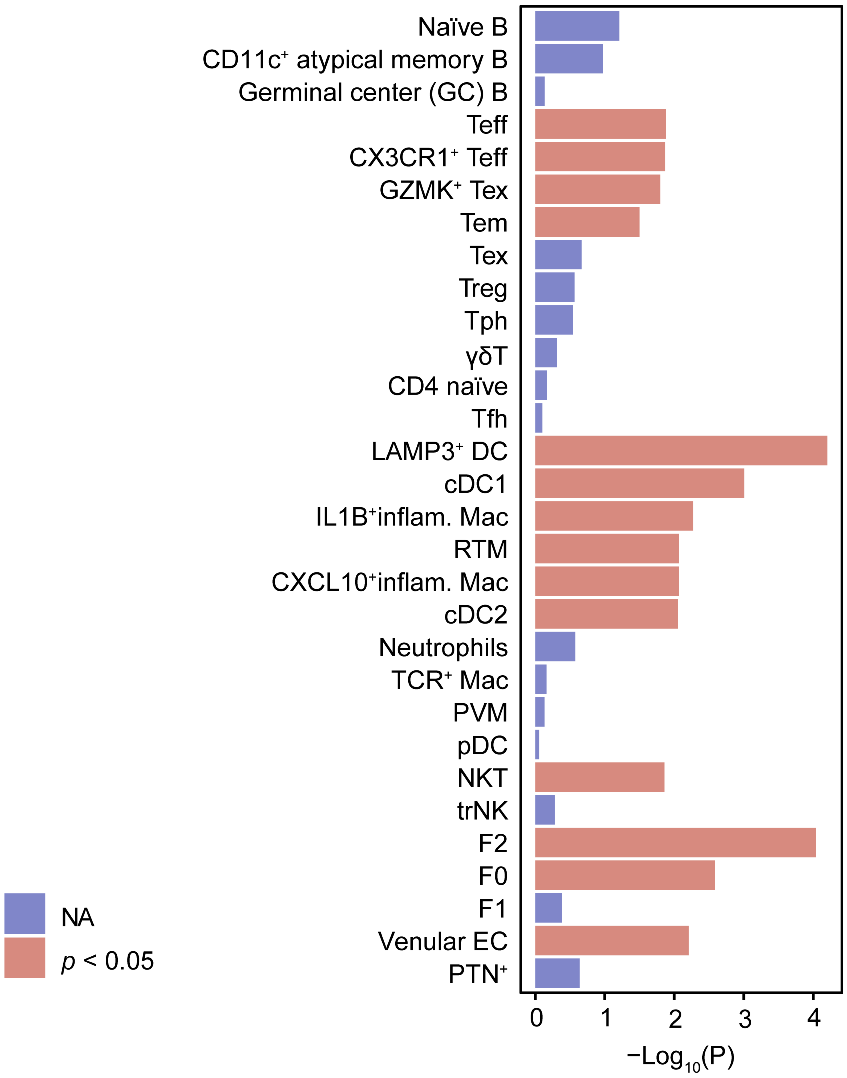

Figure S9. Cell types Relevant to the genetic risk loci of GD. Bar plots showing cell subsets associated with 44 genes from 28 previously reported GD risk loci, as identified by MAGMA. For clarity, only cell types exhibiting differential expression of at least two genes are shown.

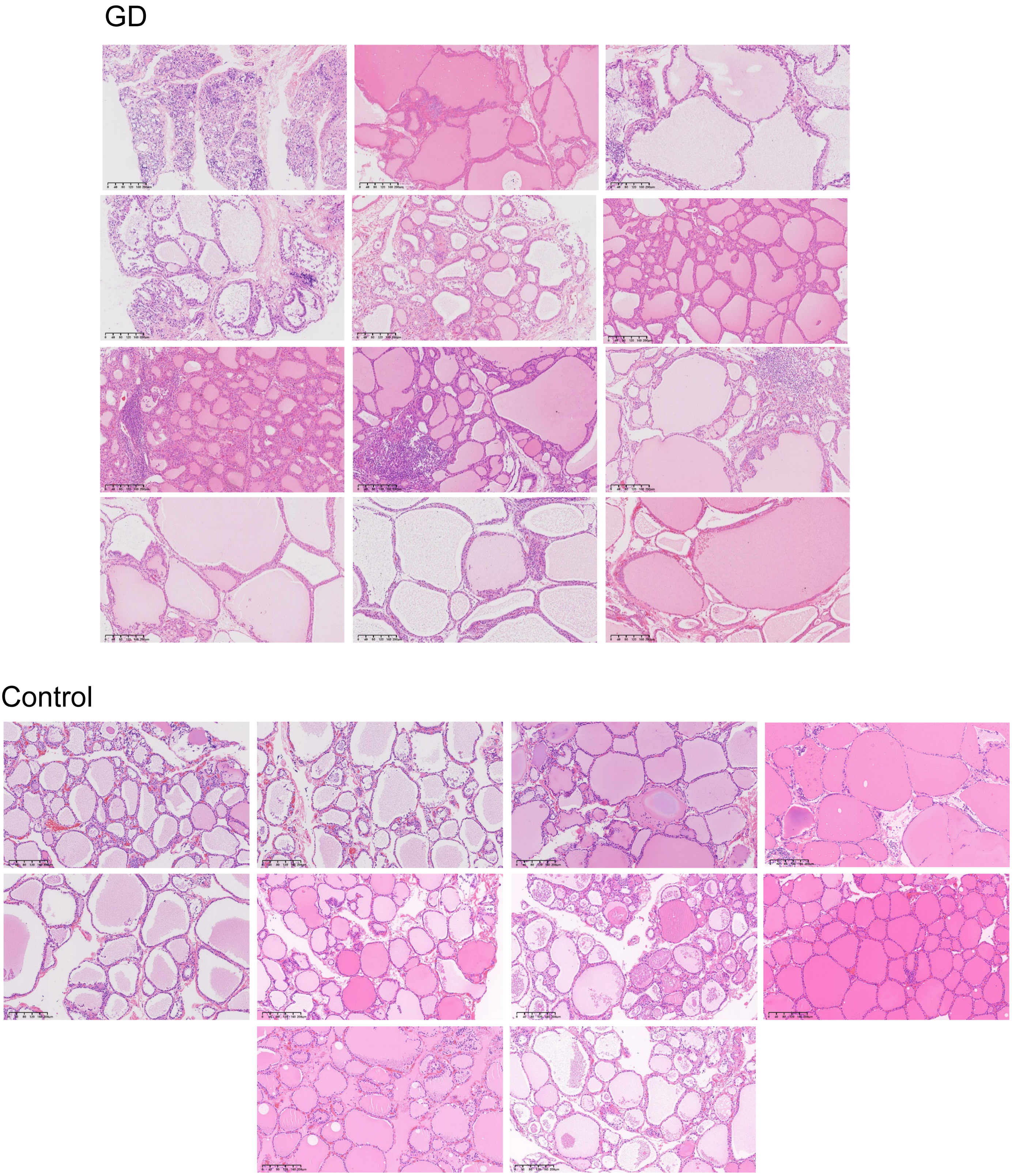

Figure S10. Hematoxylin and eosin staining of thyroid tissues from 12 GD and 10 control patients. Bar = 200 μm.
